# Switching mechanism from AR to EGFR signaling via 3-*O*-sulfated heparan sulfate in castration-resistant prostate cancer

**DOI:** 10.1038/s41598-023-38746-x

**Published:** 2023-07-18

**Authors:** Hayato Ota, Hirokazu Sato, Shuji Mizumoto, Ken Wakai, Kei Yoneda, Kazuo Yamamoto, Hayao Nakanishi, Jun-Ichiro Ikeda, Shinichi Sakamoto, Tomohiko Ichikawa, Shuhei Yamada, Satoru Takahashi, Yuzuru Ikehara, Shoko Nishihara

**Affiliations:** 1 Department of Bioinformatics, Graduate School of Engineering, Soka University, Tokyo, Japan; 2 Department of Pathobiochemistry, Faculty of Pharmacy, Meijo University, Nagoya, Aichi Japan; 3 Department of Urology, Graduate School of Medicine, Chiba University, Chiba, Japan; 4 Graduate School of Medicine, Chiba University, Chiba, Japan; 5 Laboratory of Pathology and Clinical Research, Aichi Cancer Center Aichi Hospital, Nagoya, Aichi Japan; 6 Department of Diagnostic Pathology, Graduate School of Medicine, Chiba University, Chiba, Japan; 7 Department of Experimental Pathology and Tumor Biology, Graduate School of Medical Sciences, Nagoya City University, Nagoya, Aichi Japan; 8 Department of Pathology, Graduate School of Medicine, Chiba University, Chiba, Japan; 9 Glycan & Life System Integration Center (GaLSIC), Soka University, Tokyo, Japan

**Keywords:** Glycobiology, Glycosylation

## Abstract

Androgen deprivation therapy is given to suppress prostate cancer growth; however, some cells continue to grow hormone-independently as castration-resistant prostate cancer (CRPC). Sulfated glycosaminoglycans promote ligand binding to receptors as co-receptors, but their role in CRPC remains unknown. Using the human prostate cancer cell line C4-2, which can proliferate in hormone-dependent and hormone-independent conditions, we found that epidermal growth factor (EGF)-activated EGFR–ERK1/2 signaling via 3-*O*-sulfated heparan sulfate (HS) produced by HS 3-*O*-sulfotransferase 1 (HS3ST1) is activated in C4-2 cells under hormone depletion. Knockdown of *HS3ST1* in C4-2 cells suppressed hormone-independent growth, and inhibited both EGF binding to the cell surface and activation of EGFR–ERK1/2 signaling. Gefitinib, an EGFR inhibitor, significantly suppressed C4-2 cell proliferation and growth of a xenografted C4-2 tumor in castrated mouse. Collectively, our study has revealed a mechanism by which cancer cells switch to hormone-independent growth and identified the key regulator as 3-*O*-sulfated HS.

## Introduction

Prostate cancer is a very common cancer among men, especially in the United States^1^. Normally, it is proliferated by hormones such as testosterone, which bind to androgen receptor (AR) and promote transcription in the nucleus in a hormone-dependent manner^2^. Androgen deprivation therapy (ADT), such as castration, is thus performed to suppress the growth of tumors, but some cancer cells are resistant to ADT and grow in a hormone-independent manner. Prostate cancer that is resistant to ADT is called castration-resistant prostate cancer (CRPC), and reliable treatment for CRPC has not been established. In CRPC, hormone-independent growth proceeds via enhancement of AR-independent bypass signaling—in particular, epidermal growth factor receptor (EGFR) signaling. Notably, EGFR is more enriched in hormone-insensitive than in hormone-sensitive prostate cancer cell lines^3,4^. Furthermore, the expression of activated ERK1/2, the downstream molecule of EGFR, is elevated in metastatic CRPC samples from patients^5,6^. However, the detail mechanism underlying AR-independent signal enhancement through EGFR–ERK1/2 in hormone-independent growth remains largely unclear.

Signal transduction is triggered by binding between receptor and ligand molecules on the cell membrane. A co-receptor may also be required for the ligand molecule to bind to the receptor. Among various glycans that exist on the cell surface, glycosaminoglycans (GAGs), including heparan sulfate (HS), chondroitin sulfate (CS), dermatan sulfate (DS), keratan sulfate (KS), and hyaluronan (HA), play vital roles in signal transduction as co-receptors for ligand molecules.

Among the different GAGs, HS regulates signal transduction in different cell types by binding to ligands such as fibroblast growth factor (FGF), Wnt, bone morphogenetic protein 4 (BMP4) and Hedgehog^7–10^. HS comprises a disaccharide repeating structure of *N*-acetyl-glucosamine (GlcNAc) and glucuronic acid (GlcA), which is produced in vivo through the function of Exostosins (EXTs) and EXT-like proteins (EXTLs)^11^. Sulfotransferases transfer sulfate groups to these repeating disaccharide units in a stepwise manner^12^. In the first step, the disaccharide unit undergoes *N*-deacetylation of GlcNAc, followed by *N*-sulfation by *N*-deacetylase/*N*-sulfotransferases (NDSTs). Next, some of the GlcA moieties are isomerized to IdoA by GlcA C5-epimerase (GLCE). Heparan sulfate 2-*O*-sulfotransferase (HS2ST) then transfers a sulfate group onto the C2 position of GlcA and IdoA, while sulfation of the C6 position of *N*-sulfated glucosamine (GlcNS) is catalyzed by HS 6-*O*-sulfotransferases (HS6STs). Lastly, 3-*O*-sulfation of GlcNS, the terminal modification of HS, is catalyzed by HS 3-*O*-sulfotransferases (HS3STs)^13^. In general, the different HS structures can be identified and quantified by structural analysis such as high-performance liquid chromatography (HPLC); however, 3-*O*-sulfation can interfere with HS degradation by heparin lyases, leading to the formation of lyase-resistant saccharides^14–17^. Therefore, identification and quantification of HS structures with 3-*O*-sulfation remains difficult. 3-*O*-sulfated HS (3-OS HS) plays a role in anticoagulant activity by binding to antithrombin III and is also involved in mediation of herpes virus infection^18–20^.

In this study, human prostate cancer cell line C4-2, which expresses AR and can proliferate under both hormone-dependent and hormone-independent culture conditions, was used to clarify the molecular mechanism by which cancer cells switch from hormone-dependent growth to the hormone-independent growth of CRPC^21^. We identified a novel hormone-independent growth mechanism based on EGFR–ERK1/2 signaling that requires 3-*O*-sulfation of HS catalyzed by HS3ST1. Notably, EGF activates this EGFR–ERK1/2 signaling pathway by binding to HS via 3-*O*-sulfation, although it does not have a heparin binding (HB) domain like HB-EGF^22^. Furthermore, xenografted tumor experiments with anticancer agents showed that combined castration and hormone depletion via inhibition of the EGFR–ERK1/2 pathway is highly effective for treatment of CRPC. This study demonstrates that a 3-*O*-sulfation-mediated signaling mechanism regulates switching from hormone-dependent growth to hormone-independent growth in CRPC.

## Results

### Hormone-dependent and -independent growth of the human prostate cancer cell line C4-2

**Figure 1:**
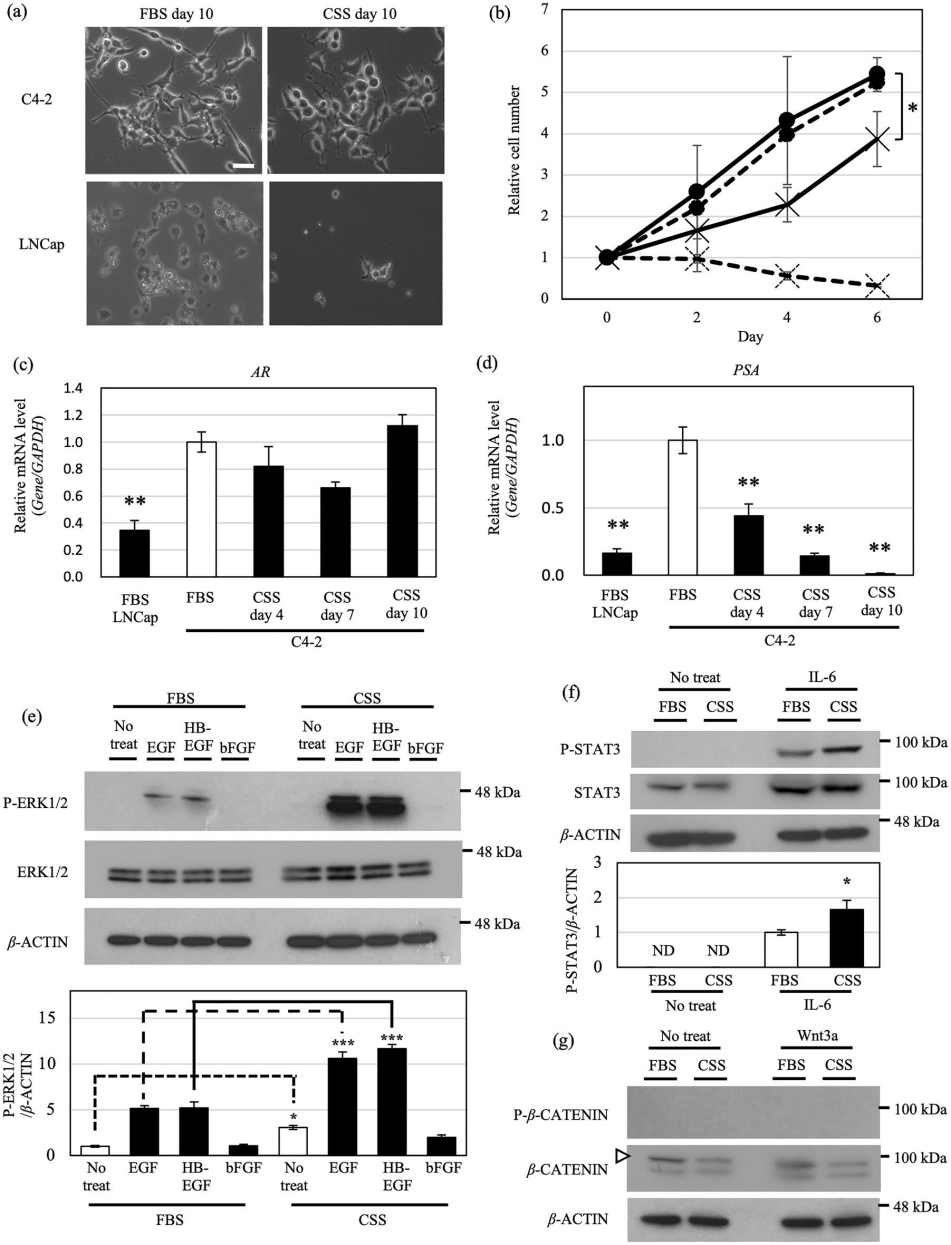
C4-2 cells proliferate via EGF and HB-EGF signaling in hormone-depleted culture conditions. (**a**) Morphology of C4-2 and LNCap cells cultured in medium containing FBS or CSS for 10 days. Representative images are shown. Scale bar, 50 μm. (**b**) Cell proliferation assay of C4-2 and LNCap cells cultured in medium containing FBS or CSS. C4-2 and LNCap cells are indicated as solid and dotted lines, respectively. FBS and CSS conditions are indicated as (●) and (×), respectively. (**c**, **d**) mRNA levels of *AR* and *PSA* in LNCap cells cultured in medium containing FBS, and C4-2 cells cultured in medium containing FBS or CSS (day 4, 7, 10) were analyzed by real-time PCR and normalized to *GAPDH* mRNA in the same sample. Expression levels are shown relative to those of C4-2 cells in FBS-containing medium. (**e**) Western blot analysis of ERK1/2 phosphorylation after growth factor stimulation for 7.5 min. C4-2 cells were cultured in medium containing FBS or CSS for 10 days before stimulation with 1 ng/ml of each growth factor (EGF, HB-EGF or bFGF). Bottom panel shows quantification of phosphorylated ERK1/2 (P-ERK1/2) in top panel normalized to *β*-ACTIN. Expression levels are shown relative to non-treated samples in FBS-containing medium. Each blot has been cropped from different gels; uncropped gels/blots are presented in Supplementary Fig. S9. (**f**) Western blot analysis of STAT3 phosphorylation after stimulation with IL-6 (10 ng/ml) for 7.5 min. Bottom panel shows quantification of phosphorylated STAT3 (P-STAT3) in top panel normalized to *β*-ACTIN. Expression levels are shown relative to IL-6-stimulated samples in FBS-containing medium. Each blot has been cropped from different gels; uncropped gels/blots are presented in Supplementary Fig. S9. (**g**) Western blot analysis of *β*-CATENIN phosphorylation after Wnt3a (10 ng/ml) stimulation for 7.5 min. Arrowhead indicates the target band corresponding to *β*-CATENIN. P-*β*-CATENIN, phosphorylated *β*-CATENIN. Each blot has been cropped from different gels; uncropped gels/blots are presented in Supplementary Fig. S9. Ratios are given as mean ± S.E. of three independent experiments. Statistical significance assessed by Student’s *t*-test or Dunnett’s test are indicated with *(*p* < 0.05), **(*p* < 0.01) or ***(*p* < 0.001). ND, not detected.

We selected C4-2 cells for this study because they can proliferate under both hormone-dependent and hormone-independent culture conditions. To confirm this, C4-2 cells and the parental LNCap cell line, which undergoes only hormone-dependent proliferation, were cultured in fetal bovine serum (FBS; hormone-dependent) and charcoal stripped serum (CSS; hormone-independent) containing medium for 10 days, and their growth was examined (Fig. 1a, b). LNCap cells proliferated in FBS-containing medium, but mostly died in CSS-containing medium. By contrast, C4-2 cells proliferated in both types of medium (Fig. 1a, b). There was no significant difference in proliferation ability between C4-2 cells and LNCap cells in FBS-containing medium; however, the growth rate of C4-2 cells in CSS-containing medium was slower than that in FBS-containing medium.

Prostate cancer specific antigen (PSA) is both a target of AR signaling and a biomarker of prostate cancer^23^. We therefore evaluated the expression levels of *AR* and *PSA* mRNAs in C4-2 cells after 10 days of culture in FBS- and CSS-containing medium. Whereas the expression of *AR* in C4-2 cells in both media did not change, that of *PSA* decreased dramatically in CSS-containing medium after 10 days (Fig. 1c, d). When the C4-2 cells cultured in CSS-containing medium were switched to FBS-containing medium for a further 24 days. (Supplementary Fig. S1a, b). *AR* was stably expressed after day 10. *PSA* was significantly increased on all days relative to day 0, as long as AR was expressed, indicating that the AR signaling pathway was reactivated. Collectively, these findings show that switching between medium containing FBS and CSS can regulate AR signal activation in C4-2 cells. Furthermore, C4-2 cells can proliferate in CSS-containing medium even though AR signaling is not activated, suggesting that they grow in CSS-containing medium through another—AR-independent—signaling pathway.

### Activation of EGF and HB-EGF/ ERK1/2 signaling in C4-2 cells in the hormone-depleted condition

To investigate the intracellular signaling pathway involved in the proliferation of C4-2 cells in CSS-containing medium, we compared intracellular signaling activity after stimulation with various growth factors and ligands in C4-2 cells cultured in the two media. When cells were stimulated with EGF or HB-EGF, which both use the common receptor EGFR, the expression of phosphorylated ERK1/2 increased significantly in C4-2 cells cultured in CSS-containing medium (Fig. 1e). By contrast, when C4-2 cells were stimulated with basic FGF (bFGF), the expression of phosphorylated ERK1/2 did not change. The expression of phosphorylated STAT3 was slightly increased by stimulation with IL-6 in C4-2 cells in CSS-containing medium (Fig. 1f). Phosphorylated *β*-CATENIN was not detected in either medium, but total *β*-CATENIN expression was decreased in CSS-containing medium (Fig. 1g). Based on these observations, we hypothesized that highly activated ERK1/2 signaling by EGF and HB-EGF is the key pathway for hormone-independent growth of C4-2 cells.

**Figure 2:**
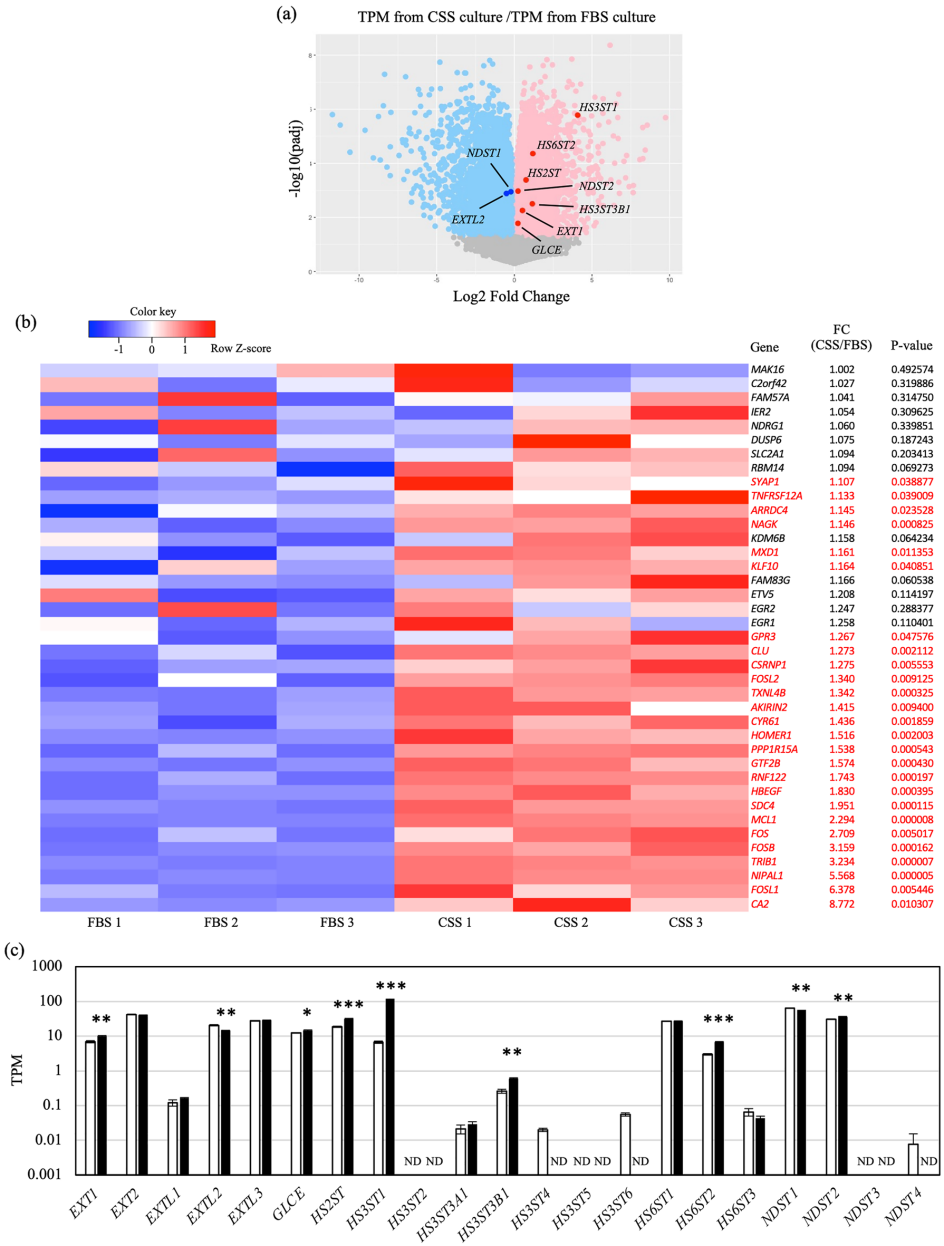
Gene expression profile from RNA-seq data. (**a**) Volcano plot of genes from RNA-seq data in C4-2 cells cultured in medium containing FBS or CSS. (**b**) Heatmap of EGFR–ERK1/2 target transcripts upregulated in C4-2 cells cultured in CSS-containing medium. Data are shown as low-expression (blue) and high-expression (red) genes. Significantly upregulated genes are named in red. Fold change (FC) was calculated by dividing TPM in CSS-containing medium by TPM in FBS-containing medium. (**c**) TPM for glycosyltransferases and sulfotransferases related to HS synthesis in C4-2 cells cultured in medium containing CSS versus medium containing FBS. Ratios are given as mean ± S.E. of three independent experiments. Statistical significance assessed by Student’s *t*-test is indicated with *(*p* < 0.05), **(*p* < 0.01) or ***(*p* < 0.001). ND, not detected.

Switching C4-2 cells from CSS- to FBS-containing medium suppressed the phosphorylation levels of activated ERK1/2 (Supplementary Fig. S1c). Furthermore, the expression of *PSA* was increased after switching from CSS- to FBS-containing medium, showing that the AR and EGFR–ERK1/2 signals could be switched, depending on the presence of hormone (Fig. 1c–e and Supplementary Fig. S1a–c). Furthermore, RNA-seq analysis revealed that the expression of some ERK target genes, including the EGFR–ERK1/2 axis target genes *FOS* and *FOSL1*, was increased in CSS-containing medium^24,25^(Fig. 2a, b). Thus, ERK target gene expression supports the activation of EGFR–ERK1/2 signaling in C4-2 cell cultured in the hormone-depleted condition. These results suggest that hormone-independent growth of C4-2 cells depends on EGFR–ERK1/2 signaling through EGF and HB-EGF instead of AR signaling. In addition, it is possible that *FOS*, which is frequently overexpressed in severe prostate tumor, and *FOSL1*, which promotes growth and metastasis of prostate cancer cells, are also involved in castration-resistance acquisition^26,27^.

### Increased expression of HS3ST1 in C4-2 cells in the hormone-depleted condition

Next, we investigated the molecular mechanism underlying EGFR–ERK1/2 activation in C4-2 cells cultured in CSS-containing medium. First, to elucidate whether the activation of EGFR–ERK1/2 signaling is triggered by an increase in ligand or receptor expression, the expression of *EGF*, *HB-EGF* and genes of the EGFR family (*EGFR*, *HER2*, *HER3* and *HER4*) was measured in C4-2 cells. *EGF* and *HB-EGF* mRNA levels did not increase in C4-2 cells cultured in CSS-containing medium (Supplementary Fig. S1d). In addition, only *EGFR* of the EGFR family was expressed in C4-2 cells, and its expression did not differ between FBS- and CSS-containing medium (Supplementary Fig. S1e). Thus, we hypothesized that the activation of EGFR–ERK1/2 signaling in hormone-independent growth is dependent on co-receptor expression.

**Figure 3:**
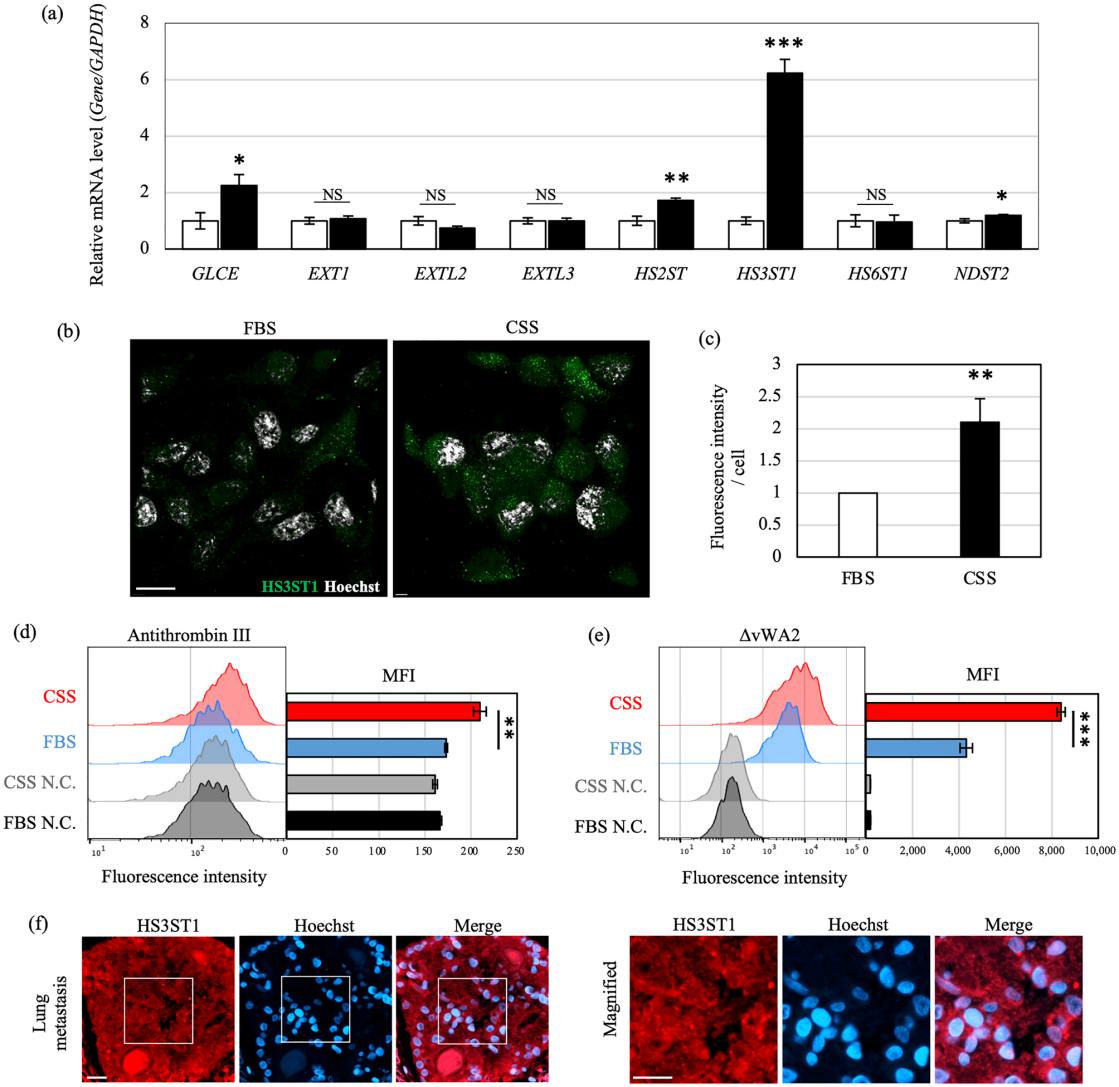
Expression of HS3ST1 is upregulated in C4-2 cells under hormone depletion. (**a**) mRNA levels of each gene were analyzed by real-time PCR and normalized to *GAPDH* mRNA in the same sample. Expression levels are shown relative to gene expression in C4-2 cells in FBS-containing medium. (**b**) Immunostaining of C4-2 cells cultured in medium containing FBS or CSS with antibody against HS3ST1 (green). Nuclei were stained by Hoechst (white). Scale bars, 20 μm. (**c**) Quantification of intensity of HS3ST1 staining in (b). Fluorescence intensity was normalized to cell number. The 3D image was created by Imaris. (**d**, **e**) Flow-cytometry analysis with antithrombin III and mutated cochlin ΔvWA2, both of which bind to 3-OS HS, in C4-2 cells cultured in medium containing FBS (blue) or CSS (red). Negative controls reacted with only secondary antibody are indicated in black and grey. Each right panel shows quantification of mean fluorescence intensity (MFI). (**f**) Immunohistochemistry in lung metastasis prostate cancer. HS3ST1 and nuclei are highlighted in red and blue, respectively. Right panel shows magnified images of the boxed regions in the left panel. Scale bars, 20 μm. Ratios are given as mean ± S.E. of three independent experiments. Statistical significance assessed by Student’s t-test is indicated with *(*p* < 0.05), **(*p* < 0.01) and ***(*p* < 0.001). NS, not significant.

The RNA-seq data showed that the expression of enzymes involved in HS elongation and sulfation was significantly changed (Fig. 2a, c). Importantly, expression of the gene encoding HS3ST1, which transfers 3-*O*-sulfation to GlcNS as an HS terminal modification when GlcA or IdoA lacks 2-*O*-sulfation^28^ (Supplementary Fig. S2a), was markedly increased in C4-2 cells cultured in CSS-containing medium (Fig. 2a, c). Among the seven *HS3ST* isoforms, *HS3ST1* expression was overwhelmingly high, indicating that 3-*O*-sulfation in C4-2 cells is mainly mediated by HS3ST1. The marked increased in expression of the *HS3ST1* gene in C4-2 cells was confirmed by real-time PCR (Fig. 3a); furthermore, the expression of HS3ST1 protein was also significantly increased (Fig. 3b, c). Conversely, when C4-2 cells were switched to FBS-containing medium, the expression of *HS3ST1* sharply decreased (Supplementary Fig. S2b). These results indicated that the increased expression of HS3ST1 was specific to the hormone-depleted condition and corresponded to a switch from AR signaling to EGFR signaling.

We also investigated the levels of the 3-OS HS structure in C4-2 cells cultured in FBS- and CSS-containing medium by using antithrombin III, which binds specifically to 3-OS HS produced by HS3ST1^18,29,30^. An antithrombin III binding assay based on flow-cytometry revealed that more antithrombin III bound the cell surface of C4-2 cells in CSS-containing medium as compared with cells in FBS-containing medium (Fig. 3d). Similarly, a greater amount of mutated cochlin (ΔvWA2), a lectin that recognizes sulfated GAGs including 3-OS HS^31^, bound to the surface of C4-2 cells in CSS-containing medium relative to FBS-containing medium (Fig. 3e). Collectively, these findings suggest that 3-OS HS produced by HS3ST1 is involved in the hormone-independent growth of C4-2 cells. In addition, we found that HS3ST1 was expressed not only in the C4-2 cell line but also in metastatic CRPC in the human lung (Fig. 3f), suggesting that this enzyme might be the key factor in CRPC progression.

### Increased 3-*O*-sulfate-containing oligosacharides of HS isolated from C4-2 cells in the hormone-depleted condition

To further confirm that the 3-OS HS structure is increased in C4-2 cells in CSS-containing medium, we compared the levels of HS structures in C4-2 cells between the two culture conditions. Because 3-*O*-sulfated GlcN (GlcNS,3S) residues in HS except for -IdoA-GlcNS,3S,6S- are resistant to bacterial heparinases, we used a combination of enzymatic treatment and anion-exchange HPLC^32^. In the resulting chromatograms, except for peaks B and F, four other fragment peaks of heparinase-resistant oligosaccharides containing 3-*O*-sulfated GlcN residues were greater in GAG–peptide fractions prepared from C4-2 cells cultured in CSS-containing medium as compared with FBS-containing medium (Supplementary Fig. S3a–f). It is likely that the increased peaks correspond to oligosaccharides derived from 3-OS HS modified by HS3ST1.

### HS3ST1-catalyzed 3-OS HS is necessary for C4-2 cell growth in the hormone-depleted condition

**Figure 4:**
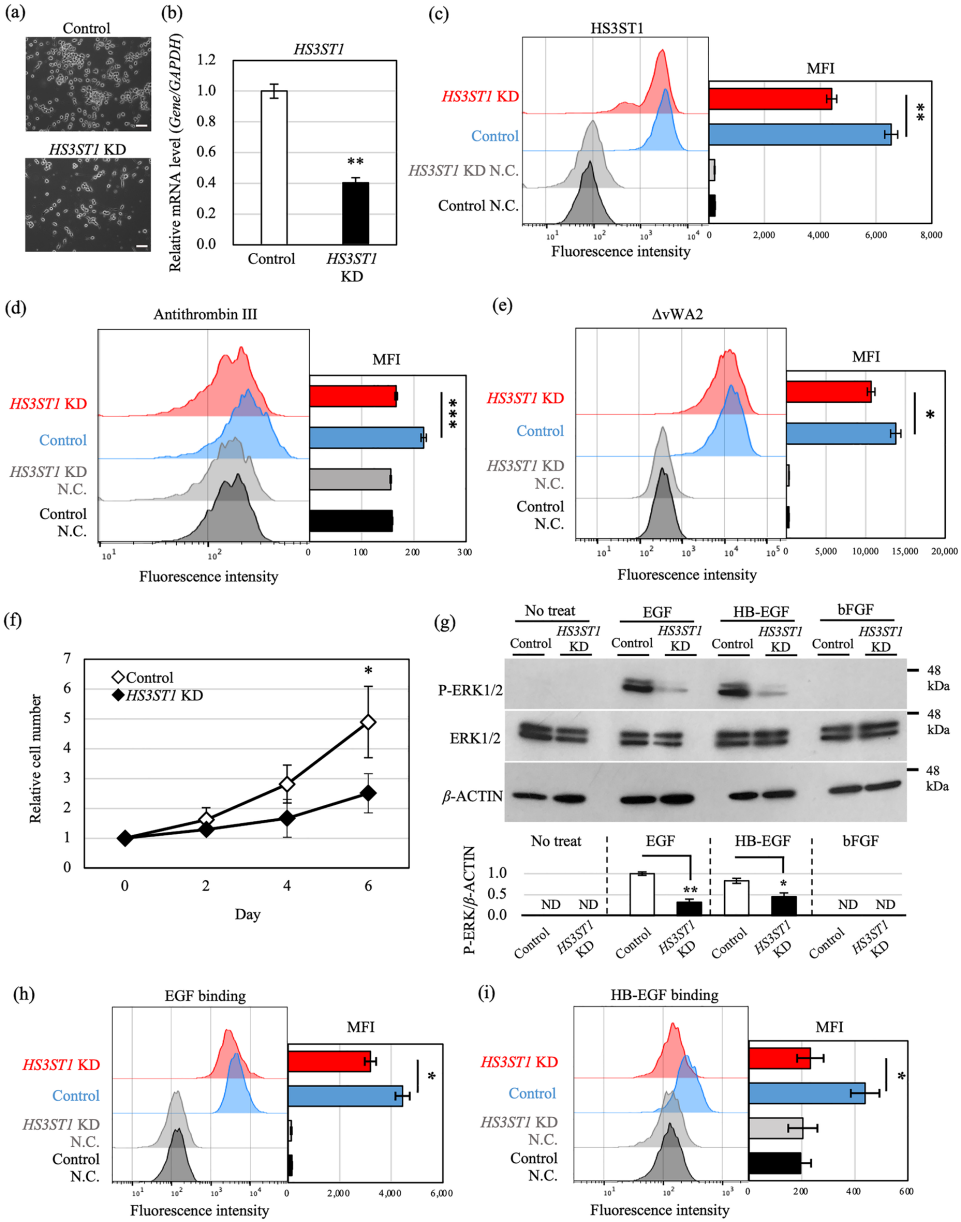
Knockdown (KD) of *HS3ST1* inhibits C4-2 cell growth mediated by EGF and HB-EGF signaling under hormone depletion. (**a**) Morphology of *HS3ST1* KD C4-2 cells in medium containing CSS. C4-2 cells were transfected with *HS3ST1* siRNA, siRNA-1, or control siRNA three times every 3 days and cultured for 9 days in total. Scale bars, 100 μm. (**b**) Knockdown efficiency of siRNA-1 was analyzed by real-time PCR. mRNA levels were normalized to *GAPDH* mRNA and are shown relative to the control. (**c**) Flow-cytometry analysis of HS3ST1 protein expression in *HS3ST1* KD C4-2 cells in medium containing CSS. Intracellular staining was performed with antibody against HS3ST1. Control and *HS3ST1* KD cells are indicated in blue and red, respectively. Negative controls reacted with only secondary antibody are indicated in black and grey. Right panel shows quantification of MFI. (**d**, **e**) Flow-cytometry analysis with antithrombin III and mutated cochlin ΔvWA2 in *HS3ST1* KD C4-2 cells in medium containing CSS. (**f**) Cell proliferation assay of C4-2 cells transfected with control (◇) and *HS3ST1* (◆) siRNA-1 in medium containing CSS. Assay was started on 9th day after siRNA-1 transfection. (**g**) Western blot analysis of ERK1/2 phosphorylation after stimulation with 1 ng/ml of growth factors for 7.5 min. Before ligand stimulation, cells were pre-cultured in serum-free medium for 24 h on 9th day after siRNA-1 transfection. Bottom panel is quantification of ERK1/2 phosphorylation normalized to *β*-ACTIN. Expression levels are shown relative to the control. Each blot has been cropped from different gels; uncropped gels/blots are presented in Supplementary Fig. S10. (**h**, **i**) Flow-cytometry analysis of EGF and HB-EGF binding to *HS3ST1* KD C4-2 cells cultured in medium containing CSS. Representative histograms from three independent experiments are shown. Control and *HS3ST1* KD cells are indicated in blue and red, respectively. Negative controls with no treatment are indicated in black and grey. Ratios are given as mean ± S.E. of three independent experiments. Statistical significance assessed by Student’s t-test is indicated with *(*p* < 0.05), **(*p* < 0.01) and ***(*p* < 0.001). ND, not detected.

Next, to clarify the function of 3-OS HS produced by HS3ST1 in the hormone-independent growth of C4-2 cells, *HS3ST1* expression was subjected to knockdown (KD) in C4-2 cells cultured in CSS-containing medium (Fig. 4a and Supplementary Fig. S4a). We confirmed that expression of HS3ST1 was significantly reduced at both the transcriptional and protein level in KD cells (Fig. 4b, c and Supplementary Fig. S4b, c). The levels of the 3-OS HS structure on the cell surface of KD cells were investigated by antithrombin III and mutated cochlin ΔvWA2 binding assays, both of which bind to 3-OS HS^18,29–31^. Antithrombin III and ΔvWA2 binding to the cell surface was significantly reduced in *HS3ST1* KD cells, confirming that *HS3ST1* KD reduced the expression of 3-OS HS in C4-2 cells in CSS-containing medium (Fig. 4d, e and Supplementary Fig. S4d, e). Furthermore, the decrease in 3-OS HS significantly suppressed the proliferation of C4-2 cells in CSS-containing medium (Fig. 4f and Supplementary Fig. S4f.). Collectively, these observations show that 3-OS HS synthesized by HS3ST1 is required for the hormone-independent growth of C4-2 cells in CSS-containing medium.

### Downregulation of EGFR–ERK1/2 signaling in *HS3ST1* KD C4-2 cells

Next, we investigated the activation of EGFR–ERK1/2 signaling in *HS3ST1* KD C4-2 cells cultured in CSS-containing medium. Expression of phosphorylated ERK1/2 was significantly reduced in *HS3ST1* KD C4-2 cells when the cells were stimulated by EGF or HB-EGF (Fig. 4g and Supplementary Fig. S4g), implying that 3-OS HS produced by HS3ST1 is required for EGF- and HB-EGF-mediated activation of ERK1/2 signaling.

Based on the hypothesis that the HS chain containing 3-OS HS functions as a co-receptor enabling EGF and HB-EGF to bind to EGFR in C4-2 cells, we examined whether EGF and HB-EGF bind to the cell surface of live *HS3ST1* KD C4-2 cells. EGF and HB-EGF binding was decreased in *HS3ST1* KD cells, indicating that both EGF and HB-EGF bind to 3-OS HS produced by HS3ST1 on the C4-2 cell surface (Fig. 4h, i and Supplementary Fig. S4h, i). Furthermore, confocal observation of *HS3ST1* KD cells after stimulation with fluorescent dye-conjugated EGF revealed that the amount of EGF that localized to the membrane was decreased (Supplementary Fig. S5). The 3D reconstruction images supported the localization of EGF around the cells. These results demonstrate that not only HB-EGF, but also EGF binds to EGFR via 3-OS HS produced by HS3ST1, and that EGFR–ERK1/2 signaling is activated by EGF and HB-EGF in C4-2 cells under hormone depletion instead of AR signaling.

To investigate whether 3-OS HS mediates the activation of EGFR-ERK1/2 signaling in a CRPC cell line that, unlike C4-2, does not express AR, we performed *HS3ST1* gene KD in PC3 cells^33^ in the hormone-depleted condition. *HS3ST1* KD resulted in a decrease in the binding of antithrombin III, confirming a reduction in the expression of 3-OS HS (Supplementary Fig. S6a). Furthermore, the expression of phosphorylated ERK1/2 was significantly reduced in *HS3ST1* KD PC3 cells upon stimulation with EGF or HB-EGF (Supplementary Fig. S6b). These findings demonstrate that 3-OS HS plays a critical role in the activation of EGFR-ERK1/2 signaling in both PC3 cells and C4-2 cells under the hormone-depleted condition, regardless of the expression of AR.

### EGFR signaling is required for growth of CRPC tumor

**Figure 5:**
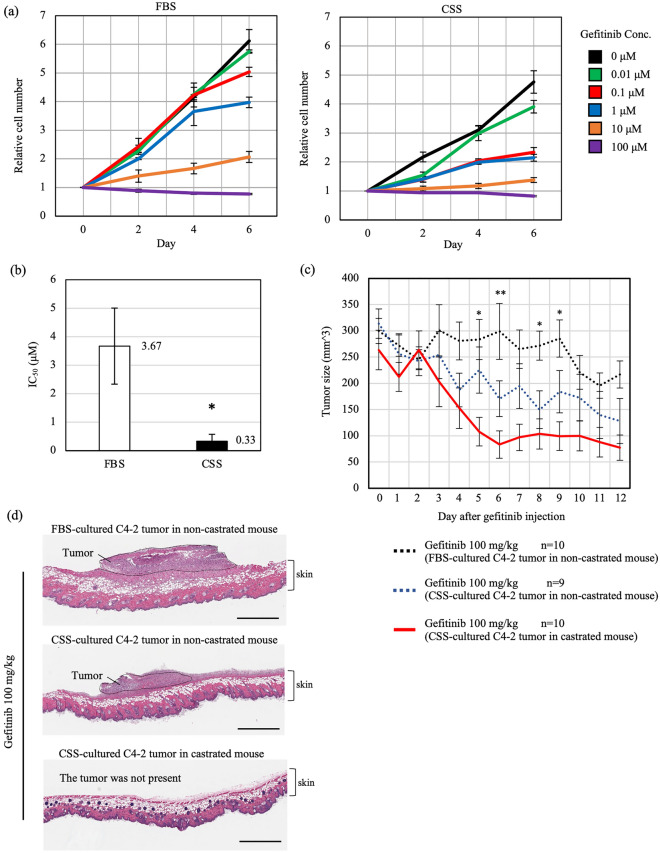
Gefitinib is effective in castrated mouse for reducing the size of xenografted tumors from C4-2 cells pre-cultured under hormone depletion. (**a**) Cell proliferation assay of C4-2 cells cultured in medium containing FBS and or CSS with gefitinib (0.01, 0.1, 1, 10, 100 μM) or DMSO as a control. Growth rate is shown relative to cell number at day 0. (**b**) IC_50_ of gefitinib on day 6 after gefitinib addition in FBS- and CSS-containing culture medium. (**c**) Effects of gefitinib on growth of a xenografted tumor in nude mice. Ratios are given as mean ± S.E. of 9 or 10 independent experiments. Statistical significance assessed by Tukey–Kramer test between gefitinib (100 mg/kg) treatment for C4-2 tumors with FBS pre-culture in non-castrated mouse (black doted line) and gefitinib (100 mg/kg) treatment for C4-2 tumors with CSS pre-culture in castrated mouse (red solid line) is indicated as *(*p* < 0.05) at days 5, 8, and 9, and **(*p* < 0.01) at day 6. (**d**) Representative image of HE staining in a xenografted tumor section with skin. Tumor sections were cut perpendicular to the skin. The region of the tumor is surrounded by a dotted line. Scale bars, 800 μm.

Lastly, we investigated the possibility of targeting EGFR signaling in the treatment of prostate tumors. To explore the inhibition of EGFR signaling in CRPC, we initially added gefitinib, an EGFR inhibitor used for the treatment of other EGFR-dependent cancers including non-small cell lung cancer, to the C4-2 cell culture medium^34,35^. C4-2 cells in FBS-containing medium showed almost no change in proliferation at concentrations of 0, 0.01, and 0.1 μM gefitinib, whereas those in C4-2 cells in CSS-containing medium showed significantly reduced proliferation on the 6th day after addition of 0.1 μM gefitinib (Fig. 5a). The IC_50_ of gefitinib in C4-2 cells in CSS-containing medium was 0.33 μM, while that in FBS-containing medium was 3.67 μM (Fig. 5b). This result further shows that the hormone-independent growth of C4-2 cells proceeds via EGFR signaling.

To examine the effect of gefitinib on prostate cancer tumors, xenograft tumorigenesis experiments using C4-2 cells cultured in FBS- or CSS-containing medium were performed in nude mice (Supplementary Fig. S7a). C4-2 cells cultured in CSS-containing medium were injected into non-castrated and castrated mice, whereas C4-2 cells cultured in FBS-containing medium were injected into only non-castrated mice. All mice were then treated with gefitinib at 50 or 100 mg/kg body weight after the tumors had grown to a sufficient size. We measured tumor size every day and harvested tumors on 12th day (Fig. 5c, Supplementary Fig. S7b and Supplementary Table S1). To provide further support for the observed differences in tumor size between the groups, we used hematoxylin and eosin (HE) staining to observe sections of the tumors dissected at day 12 (Fig. 5d and Supplementary Fig. S7c). On the 12th day after administration of 100 mg/kg gefitinib, the tumors in castrated mice derived from C4-2 cells cultured in CSS-containing medium were much smaller than the tumors in non-castrated mice derived from C4-2 cells cultured in FBS-containing medium (Fig. 5c, d). Even in non-castrated mice, gefitinib was more effective for tumors derived from C4-2 cells cultured in CSS-containing medium than for tumors derived from C4-2 cells cultured in FBS-containing medium. Furthermore, among tumors derived from C4-2 cells cultured in CSS-containing medium, gefitinib was more effective in castrated mice than in non-castrated mice. The HE-stained tumor sections highlighted the pronounced impact of gefitinib treatment on tumor growth in the hormone-independent context (Fig. 5d). These findings indicate that the antitumor effect of gefitinib is more effective for reducing prostate tumors in a hormone-depleted environment than for those where hormone is present, further demonstrating that the hormone-independent proliferation of CRPC is dependent on EGFR signaling.

## Discussion

**Figure 6:**
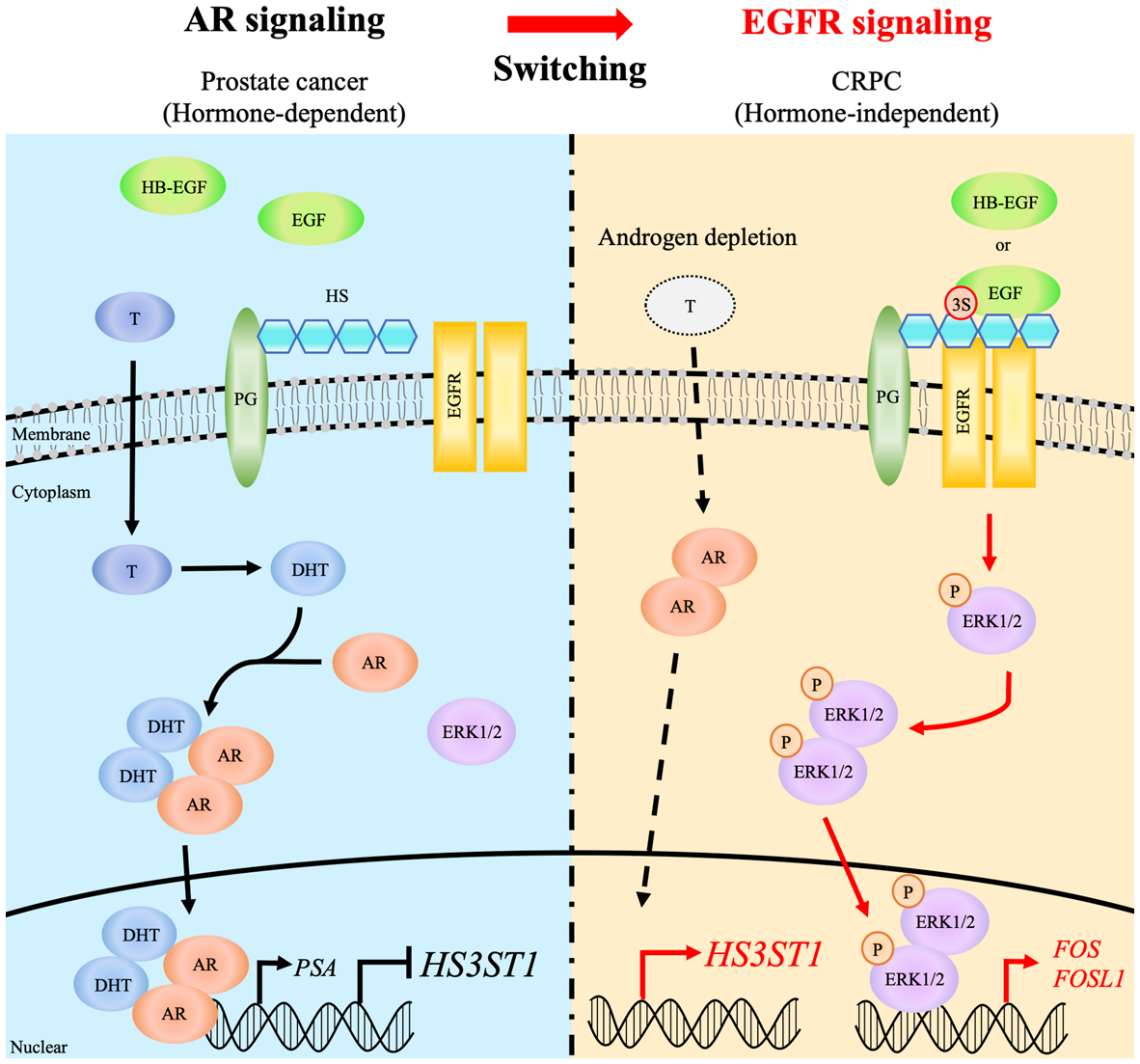
Representation of 3-OS HS function in hormone-independent prostate cancer growth. In the presence of androgen, prostate cancer cells proliferate through the activation of AR signaling. The expression of HS3ST1 and its product, 3-OS HS, is upregulated after androgen depletion. EGF and HB-EGF activate EGFR–ERK1/2 signaling by binding to EGFR via 3-OS HS. The activated EGFR–ERK1/2 signaling pathway promotes the growth of prostate cancer cells instead of AR signaling. T, testosterone; DHT, dihydrotestosterone; PG, proteoglycan.

In this study, we have elucidated the mechanism by which AR signaling switches to EGFR signaling in the growth of CRPC under hormone deprivation (Fig. 6). Androgens such as testosterone and dihydrotestosterone activate AR signaling to promote the proliferation of prostate cancer cells. The expression of HS3ST1 and 3-OS HS is suppressed when AR signaling is activated. In the case that the cancer cells acquire resistance to ADT as CRPC, however, the expression of HS3ST1 and 3-OS HS is upregulated, facilitating the binding of EGF and HB-EGF to EGFR via 3-OS HS. As a result, CRPC growth is promoted under conditions of hormone depletion, and 3-OS HS produced by HS3ST1 is the key factor in activating EGFR signaling in this growth.

To mimic the environment of prostate tumors, we explored the C4-2 cell line as a CRPC model in FBS- or CSS-containing culture medium. In C4-2 cells cultured in CSS-containing medium, reduced expression of *PSA*, a target gene of AR signaling, and EGF- and HB-EGF-mediated activation of phosphorylated ERK1/2 were observed. These results demonstrate that AR signaling is suppressed and EGFR signaling is activated in CRPC. Conversely, switching back from CSS- to FBS-containing medium suppressed EGFR signaling and restored *PSA* expression. These observations suggest that AR signaling inhibits EGFR signaling.

Regarding prostate cancer, the crosstalk between AR and EGFR signaling has been discussed from different viewpoints depending on the cell lines and culture conditions. EGF–EGFR signaling is reported to repress the expression of AR and *PSA* in both hormone-dependent and hormone-independent cell lines^36–38^. On the other hand, in hormone-independent VCap cells, EGFR, AR, and Matrix Metalloproteinase-9 (MMP-9) are reported to cooperate to promote cancer progression^39^. No consensus about the crosstalk between AR and EGFR signaling has been reached. Our study has now identified an HS3ST1-mediated mechanism that switches from AR signaling to EGFR signaling in CRPC. Consistent with the EGFR signal inhibition by AR signaling, the expression of *HS3ST1*, which increases 3-*O*-sulfation on HS and promotes EGFR signaling, was markedly increased, while *PSA* expression was decreased in C4-2 cells cultured in CSS-containing medium. Furthermore, changing back from CSS- to FBS-containing medium restored the expression of *PSA* and repressed the expression of *HS3ST1*. Thus, the switching between AR and EGFR signaling is reversible, depending on the presence of hormones.

AR is known to function as a transcriptional repressor together with specific molecules such as EZH2, the core component of the chromatin remodeler polycomb repressive complex 2 (PRC2), in addition to activating the transcription of target genes^40–43^. Our recent study showed that the expression of several glycosyltransferases is regulated by PRC2 in mouse embryonic stem cells; therefore, we also explored whether glycosyltransferases and sulfotransferases involved in HS synthesis are regulated by specific molecules in prostate cancer cells by using data in ChiP-Atlas^44,45^. We extracted ChiP-seq data in ethanol for the hormone-depleted condition and data in mibolerone, an anabolic–androgenic steroid, to represent the hormone-present condition^46,47^ (Supplementary Fig. S8a). The data show that AR binds to the genomic regions of glycosyltransferase and sulfotransferase genes involved in HS synthesis in C4-2 cells. Furthermore, there seem to be three AR binding patterns: AR binds only in the presence of hormone, AR binds both in the presence of hormone and in depleted conditions, and AR does not bind in either condition. Combined with our RNA-seq data from C4-2 cells cultured in FBS- and CSS-containing medium, genes related to HS synthesis can also be divided into three groups: AR-repressed genes, AR-activated genes, and AR-independent genes (Supplementary Fig. S8a). Furthermore, ChiP-Atlas shows that EZH2 binds to the *HS3ST1* genomic region in LNCap cells^48^. Collectively, these findings suggest that *HS3ST1* transcription is repressed by AR binding to the *HS3ST1* genomic region along with EZH2, and this may regulate the switch between AR and EGFR signaling.

Knockdown of the *HS3ST1* gene in C4-2 cells revealed that the 3-OS HS structure synthesized by HS3ST1 activates EGFR signaling and promotes proliferation in the absence of hormones. Importantly, in addition to HB-EGF, which is known to bind strongly to HS, EGF was indicated to activate EGFR signaling by binding to the 3-OS HS structure synthesized by HS3ST1. In fact, EGF binding to C4-2 cells was reduced by *HS3ST1* KD, even though it has been previously reported that EGF does not bind to HS owing to its lack of a heparin-binding domain^49–52^. However, one study has reported that 3-*O*-sulfation by HS3ST2 traps growth factors including EGF and inhibits signal activation in ovarian cancer, although the interaction has not been shown in direct binding experiments^53^. Nevertheless, it supports our observation that EGF can bind to HS, especially 3-*O*-sulfated HS. Furthermore, our study has shown that the strong binding between HB-EGF and HS is also regulated by 3-*O*-sulfation.

Some studies have reported different functions of HS with varying sulfation patterns in hormone-independent prostate cancer cells cultured in FBS-containing medium. In C4-2B cells, for example, HS2ST, which catalyzes 2-*O*-sulfation, promotes cell proliferation and invasion^54^. By contrast, SULF2, which removes 6-*O*-sulfation from GlcN(NS), promotes cell survival and cell migration in DU145 and PC3 cells, indicating that 6-*O*-sulfation suppresses the tumorigenicity of CRPC^55^. Thus, the function of HS is dependent on its sulfation pattern. Our data now demonstrate the importance of a rare terminal modification of HS, 3-*O*-sulfation, in the growth of CRPC.

The presence of core protein is also important for HS function. The RNA-seq data of HS proteoglycan (HSPG) genes in C4-2 cells cultured in FBS- and CSS-containing medium showed that 14 genes were upregulated in the hormone-depleted condition (Supplementary Fig. S8b). These HSPGs might be candidates for core proteins of 3-*O*-sulfated HS catalyzed by HS3ST1. Among them, syndecan-1, glypican-1 and perlecan have been well studied in hormone-independent prostate cancer cell lines^56–58^. Syndecan-1 is also associated with the progression and chemotherapy resistance of CRPC tumors in patients^59,60^. Because the function of HSPGs is affected by the sulfation pattern of HS, the results of our study will also contribute to clarification of the role of HSPGs in prostate cancer.

In the xenografted tumor experiments, gefitinib was more effective in the absence of hormones, owing to the inverse correlation between AR signaling activity and EGFR signaling activity associated with HS3ST1 expression. The experiments also showed that the switch between AR signaling and EGFR signaling is mediated by HS3ST1 in tumor formation and growth. To date, docetaxel and cabazitaxel, both taxane-based chemotherapeutic agents, have been commonly used for CRPC treatment^61,62^. However, taxane-based chemotherapeutic agents bind to microtubules and stabilize microtubule polymerization, as well as inhibiting cell division in CRPC. Because the effects on microtubules also affect normal cells, taxane-based therapeutic drugs generally have strong side effects for patients. This study has now shown that gefitinib, a molecularly targeted drug with fewer side effects, is effective for CRPC treatment. By administering gefitinib together with hormone-depleted therapy for prostate cancer, it is expected that the switch to EGFR signaling via 3-*O*-sulfated HS will be suppressed and the development of CRPC will be prevented.

This study focused on the sulfation of cell surface HS and its role. In xenograft experiments, Matrigel is commonly used as a tumor scaffold; however, Matrigel contains HS with a poorly defined structure that can vary between lots. We considered that if we used Matrigel as a tumor scaffold, we would not be able to study the effects of the specific cell surface HS structure. Therefore, we chose not to use Matrigel, and consequently, the xenografted tumors did not proliferate sufficiently, leading to the differences observed in the proliferation rates of C4-2 cells between in vitro and in vivo conditions. In addition, there may be differences in the effect of castration among the mice, and no experiments were conducted to confirm the amount of hormone in castrated mouse blood.

Lastly, IL-6/STAT3 signaling may also contribute to the growth of C4-2 cells cultured in medium containing CSS as we found that IL-6/STAT3 signaling is elevated in C4-2 cells cultured in CSS-containing medium. IL-6/STAT3 signaling has been previously reported as a key modulator of CRPC progression^63^.We previously reported that, in mouse ES cells, a GalNAcβ1-4GlcNAc (LacdiNAc) structure on the LIF receptor and gp130 are required to activate LIF/STAT3 signaling^64^. Because IL-6 also binds to gp130 to activate downstream signaling, the LacdiNAc structure might also be involved in IL-6/STAT3 signaling activation in CRPC. The function of LacdiNAc in prostate cancer will be analyzed in future studies.

In summary, we have revealed a mechanism by which prostate cancer cells switch from hormone-dependent growth mediated through AR signaling to hormone-independent growth mediated through EGFR signaling; in addition, we have identified the key regulator as 3-*O*-sulfated HS produced by HS3ST1. These findings will contribute to the future treatment of prostate cancer and CRPC.

## Methods

### Cell culture

The human prostate cancer cell lines LNCap and C4-2 were grown under hormone-dependent and hormone-independent conditions. For hormone-dependent growth, cells were maintained in RPMI medium (Gibco) containing 10% FBS (Gibco) and 1% penicillin/streptomycin (Invitrogen). For hormone-independent growth, 10% CSS (Invitrogen) was used instead of FBS. PC3 was cultured in CSS-containing medium. The medium was changed every 3 days, and cells were exfoliated with 0.02% EDTA and passaged every 6 days.

### Transient knockdown of *HS3ST1*

To knockdown the *HS3ST1* gene, two KD sequences, 10 nM Stealth siRNA oligo (siRNA-1; HSS115087, Invitrogen) and 10 nM Silencer Select siRNA oligo (siRNA-2; s19336, Invitrogen) were respectively transfected into C4-2 cells with Lipofectamine 2000 (Invitrogen). Stealth RNAi Negative Control Low GC Duplex # 2 (Invitrogen) was used as a negative control for siRNA-1 and Silencer™ Select Negative Control No. 1 siRNA (Invitrogen) as a negative control for siRNA*-*2. siRNA-1 was transfected into cells three times every 3 days, and cells were collected on the 9th day. siRNA-2 was transfected five times every 3 days, and cells were collected on the 15th day. For PC3, siRNA-1 was transfected once, and cells were collected on the 3rd day.

### Real-time PCR and RNA sequencing

Total RNA was isolated from cells using TRIzol Reagent (Invitrogen) and reverse transcribed using Oligo dT primer (Invitrogen) and SuperscriptII First Strand Synthesis kit (Invitrogen). Real-time PCR was performed using Quant Studio 12K Flex (Applied Biosystems) using the primers listed in Supplementary Table S2.

The relative amount of each mRNA was normalized to the amount of *GAPDH* mRNA in the same sample. RNA-seq libraries were prepared from total RNA using the TruSeq Stranded mRNA Prep kit (Illumina) according to the manufacturer’s instructions. Sequencing of 100-bp single reads was performed using a NovaSeq 6000 sequencer (Illumina). RNA-seq data were analyzed, and the heatmap and volcano plot were created with RStudio^65^.

### Cell proliferation assay

Proliferation assay of *HS3ST1* KD cells was started on 9th day after siRNA-1 transfection or 15th day after siRNA-2 transfection. For cell proliferation assay, Cell Counting Kit-8 (Dojindo) was used according to manufacturer’s protocol at 0, 2, 4, and 6 days. The reaction was performed for 1 h under 5% CO_2_. The absorbance at 450 nm of supernatant was measured with a microplate reader (Thermo Scientific). In some cases, gefitinib (Santa Cruz Biotechnology) was added when cells were seeded in each well and the measurement of absorbance was performed in the same manner. DMSO was used as a control.

### Western blot analysis

C4-2 cells were solubilized with a solubilizing solution (1% SDS) and denatured at 99 °C. Samples were separated on an SDS–polyacrylamide gel and transferred to a PVDF membrane (Millipore). Membranes were blocked with 1% BSA and incubated with primary antibodies at 4 °C overnight, followed by secondary antibody at room temperature for 60 min. Detection was performed with ECL prime (GE healthcare). For ligand stimulation experiments, C4-2 cells were precultured in serum-free medium for 24 h, and then each ligand listed in Supplementary Table S3a in serum-free medium was added to the cells. The reaction was carried out in a CO_2_ incubator for 7.5 min and cells were collected with a solubilized solution. The antibodies used for western blotting were listed in Supplementary Table S3b. For ligand stimulation to *HS3ST1* KD cells, medium was replaced with serum-free medium on 9th day after siRNA-1 transfection or 15th day after siRNA-2 transfection, respectively, and reaction was carried out 24 h later as described above.

### Flow-cytometry

2 × 10^5^ cells were suspensed in FACS buffer (0.5% BSA (Iwai), 0.1% sodium azide (Sigma-Aldrich) in PBS). The cell suspension was incubated with the antibodies or molecules in FACS buffer at 4 °C for 30 min, followed by secondary antibodies at 4 °C for 30 min. The antibodies and molecules used for flow-cytometry were listed in Supplementary Table S3c. To analyze internal HS3ST1 expression, cells were fixed and permeabilized with 100% methanol (Wako) before staining with the antibody. Samples were analyzed using a BD FACS Aria III Cell Sorter (BD Biosciences). Cells were gated to exclude debris, dead cells (identified by staining with propidium iodide; Sigma-Aldrich), and doublets.

### Immunostaining of C4-2 cells

Cells were fixed with 4% paraformaldehyde in PBS and washed with PBS. Fixed cells were blocked with 1% BSA/0.3% Triton X-100 in PBS. For primary labeling, cells were incubated with anti-HS3ST1 (14358-1-AP; Proteintech) at 4 °C overnight. Next, the cells were stained with Alexa Fluor 488-conjugated anti-rabbit IgG (A11008; Life Technologies) and Hoechst 33342 (Invitrogen). Images were obtained using an LSM 700 confocal laser microscope (Carl Zeiss).

### Confocal observation of EGF binding to cells

C4-2 cells were precultured in serum-free medium for 24 h, and then Alexa Fluor 647-conjugated human recombinant EGF (Thermo Fisher) in serum-free medium was added. The reaction was carried out for 7.5 min in a CO_2_ incubator. Cells were fixed with 4% paraformaldehyde and stained with Hoechst 33342 (Invitrogen). Images were obtained using an LSM 700 confocal laser microscope (Carl Zeiss).

### Xenografted tumor

C4-2 cells were reconstituted at 2 × 10^7^ cells in 0.2 ml of RPMI medium. Cells were subcutaneously injected into the lateral side of the right abdomen of 8-week-old male nude mice (KSN strain; Japan SLC, Inc). Castrated and non-castrated nude mice were prepared and maintained under specific pathogen-free conditions. Castrated mice were randomly divided into three groups while non-castrated mice were randomly divided into four groups. Mice were intraperitoneally injected and administered gefitinib at a dose of 0, 50, or 100 mg/kg five times a week for 2 weeks starting 10 days after cell injection. Control mice were administered 0.5% Tween 80 (Tokyo Chemical Industry) in the same manner. The maximum diameter of the tumor (L) and the diameter perpendicular to its axis (W) were measured daily. Measurements were performed until the tumors in half of the gefitinib groups disappeared. The tumor volume was calculated by the formula (L × W^2^)/2. In each group, 9–10 tumors were evaluated. The tumors that developed were harvested on the 12th day after gefitinib administration and bisected. One half was prepared as tissue sections together with the skin; the other half was carefully separated from the skin and weighed. The measured weights of the tumors were multiplied by two and are reported in Supplementary Table S1.

All protocols involving mice followed the Guide for the Care and Use of Laboratory Animals, and were approved by the Animal Care and Use Committee of Soka University. This study is reported in accordance with ARRIVE guidelines.

### Tissue samples

Lung samples from a patient with prostate cancer were obtained from an autopsy with relevant named guidelines and regulations. Informed consent was obtained from the patient. The Ethics Committee of Chiba University reviewed the project for the identification and confirmation of cancer metastasis using a near-infrared imaging method in human tissue obtained from autopsy (Research subject number: 2218)^66^. Lung specimens were fixed in 10% neutral buffered formalin and embedded into paraffin.

### Immunohistochemical analysis

The detailed immunohistochemical methods have been reported previously^67,68^. In brief, immunohistochemical analysis for lung samples was performed on 4 µm sections of paraffin-embedded specimens. After deparaffinization and hydration, the slides were treated with endogenous peroxidase in 0.3% hydrogen peroxide solution in 100% methanol for 30 min, after which the sections were blocked with 1.5% blocking serum (Santa Cruz Biotechnology) in PBS before reacting with anti-HS3ST1 antibody at 4 °C in a moist chamber overnight. The slides were washed and treated with Envision reagent, followed by color development in 3,3′-diaminobenzidine tetrahydrochloride (Dako). The slides were lightly counterstained with hematoxylin, dehydrated with ethanol, cleaned with xylene, and mounted. To avoid non-specific binding, immunizing peptide blocking was performed.

Subcutaneous tumors that formed 12 days after the injection of gefitinib into male nude mice were removed and fixed in 10% buffered formalin for 24 h. Formalin-fixed and paraffin-embedded sections (4 μm) were stained with hematoxylin.

### Disaccharide composition and 3-*O*-sulfate-containing HS structures from prostate cancer cells

The levels of total disaccharides of HS in C4-2 cells were determined as described previously^69^. In brief, cells were collected using a rubber scraper, sonicated using an Ultrasonic homogenizer (Taitec), and treated exhaustively with actinase E (Kaken Pharm). The remaining proteins and peptides were precipitated with trichloroacetic acid, and then extracted with ether to remove trichloroacetic acid. The resultant crude GAG-peptide fractions were desalted by an Amicon Ultra-4 (3 K, Millipore), and treated with heparinase-I, heparinase-III (IBEX Pharmaceuticals), and heparinase-II (R&D Systems) mixture to analyze the disaccharide composition of HS and heparinase-resistant oligosaccharides, which may contain 3-*O*-sulfated GlcN residues. The digested samples were labeled with a fluorophore 2-aminobenzamide (2AB), and the resulting 2AB-derivatives of di- and oligo-saccharides were analyzed by anion-exchange HPLC on a PA-G column (YMC Co.)^69,70^. Unsaturated disaccharides in the digests were identified by comparison with the elution positions of authentic 2AB-labeled disaccharide standards and newly prepared 3-*O*-sulfate-containing oligosaccharides.

### Preparation of 3-*O*-sulfate-containing oligosaccharides from heparin

3-*O*-sulfate-containing tetrasaccharides, ∆HexA-GlcNAc(6S)-GlcA-GlcN(NS,3S) and ∆HexA-GlcNAc(6S)-GlcA-GlcN(NS,3S,6S) (where ∆HexA, GlcN, NS, 3S, and 6S are 4,5-unsaturated hexuronic acid, glucosamine, 2-*N*-sulfate, 3-*O*-sulfate, and 6-*O*-sulfate, respectively) were prepared and labeled with a fluorophore 2AB^70,71^. 3-*O*-sulfate-containing oligosaccharides were prepared from heparin as described previously^32^. In brief, commercial heparin (Nacalai tesque) was exhaustively digested with a mixture of heparinase-I, heparinase-III, and heparinase-II for 48 h at 37 °C in 20 mM sodium acetate buffer (pH 7.0) and 2 mM Ca(OAc)_2_. The digested sample was fractionated by anion-exchange HPLC on a PA-G column. The eluate was monitored by absorbance at 232 nm generated by the unsaturated bond at the non-reducing hexuronic acid. Fractions containing heparinase-resistant oligosaccharides were separately pooled, concentrated, and desalted through a Superdex Peptide column (1.0 × 30 cm, GE healthcare). Each heparinase-resistant oligosaccharide fraction was labeled with 2AB and aliquots of the 2AB-derivatives of heparinase-resistant oligosaccharides were analyzed by anion-exchange HPLC on a PA-G column^70,71^.

### Statistical analysis

Unpaired two-tailed Student’s t-test was used to compare two groups. Dunnett’s test was used to compare more than two groups against a single control group. Tukey–Kramer test was used to assess significance in the xenografted tumor experiment. Error bars represent s.e.m.

## Supplementary Information

Supplementary Information 1.

Supplementary Information 2.

Supplementary Information 3.

Supplementary Information 4.

## Data Availability

The RNA-seq data have been deposited in the National Center for Biotechnology Information Gene Expression Omnibus under accession no. GSE223471.
