# Supplementary material for: Switching mechanism from AR to EGFR signaling via 3-O-sulfated heparan sulfate in castration-resistant prostate cancer
Source: Sci Rep. 2023 Jul 18;13:11618. doi: 10.1038/s41598-023-38746-x (PMC10354070; doi:10.1038/s41598-023-38746-x)
Supplement: Supplementary file 4 — Supplementary Information 4. [file 41598_2023_38746_MOESM4_ESM.pdf]

Supplementary Information

Switching mechanism from AR to EGFR signaling via 3-*O*-sulfated heparan sulfate in castration-resistant prostate cancer

Hayato Ota[1], Hirokazu Sato[1], Shuji Mizumoto[2], Ken Wakai[3], Kei Yoneda[3], Kazuo Yamamoto[4], Hayao Nakanishi[5], Jun-Ichiro Ikeda[6], Shinichi Sakamoto[3], Tomohiko Ichikawa[3], Shuhei Yamada[2], Satoru Takahashi[7], Yuzuru Ikehara[8], and Shoko Nishihara[1, 9, *]

[1]Department of Bioinformatics, Graduate School of Engineering, Soka University, Tokyo, Japan.

[2]Department of Pathobiochemistry, Faculty of Pharmacy, Meijo University, Nagoya, Aichi, Japan.

[3]Department of Urology, Graduate School of Medicine, Chiba University, Chiba, Japan.

[4]Graduate School of Medicine, Chiba University, Chiba, Japan.

[5]Laboratory of Pathology and Clinical Research, Aichi Cancer Center Aichi Hospital, Nagoya, Aichi, Japan.

[6]Department of Diagnostic Pathology, Graduate School of Medicine, Chiba University, Chiba, Japan.

[7]Department of Experimental Pathology and Tumor Biology, Graduate School of Medical Sciences, Nagoya City University, Nagoya, Aichi, Japan.

[8]Department of Pathology, Graduate School of Medicine, Chiba University, Chiba, Japan.

[9]Glycan & Life System Integration Center (GaLSIC), Soka University, Tokyo, Japan.

*Corresponding author: shoko@soka.ac.jp

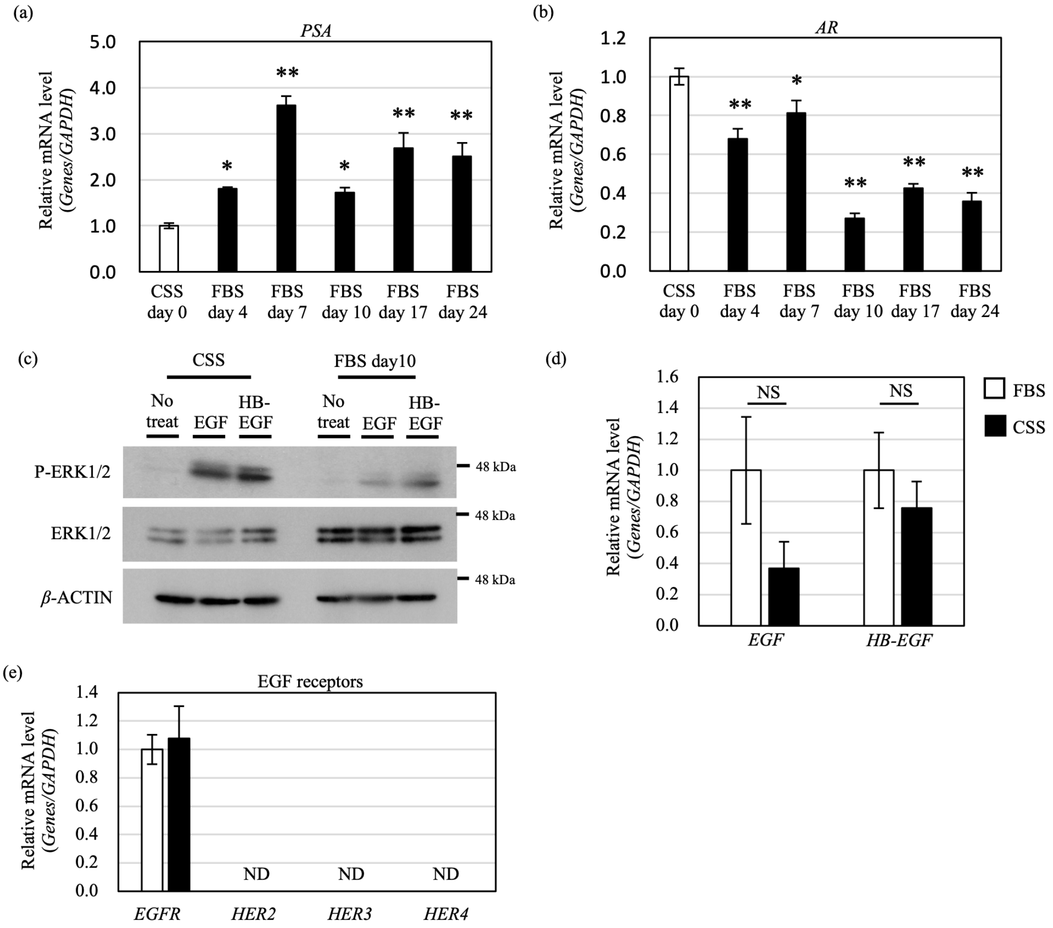

Supplementary Figure S1. AR signaling is reactivated in C4-2 cells cultured in medium containing FBS after pre-culture in CSS-containing medium. (**a**, **b**) mRNA levels of *AR* and *PSA* in C4-2 cells cultured in medium containing FBS after pre-culture in CSS-containing medium for 24 days were analyzed by real-time PCR and normalized to *GAPDH* mRNA in the same sample. Expression levels are shown relative to gene expression at day 0. (**c**) Western blot analysis of ERK1/2 phosphorylation after stimulation with 1 ng/ml of growth factor (EGF or HB-EGF) for 7.5 min. C4-2 cells were cultured in medium containing FBS after pre-culture in CSS-containing medium for 10 days. Each blot has been cropped from different gels; uncropped gels/blots are presented in Supplementary Fig. S11. (**d**, **e**) mRNA levels of *EGF, HB-EGF* and EGFR family genes in C4-2 cells cultured in medium containing FBS (white) or CSS (black) were analyzed by real-time PCR and normalized to *GAPDH* mRNA in the same sample. Expression levels are shown relative to gene expression in C4-2 cells in FBS-containing medium. Ratios are given as mean ± S.E. of three independent experiments. Statistical significance

assessed by Student's t-test or Dunnett's test is indicated with *($p<0.05$) or **($p<0.01$). ND, not detected; NS, not significant.

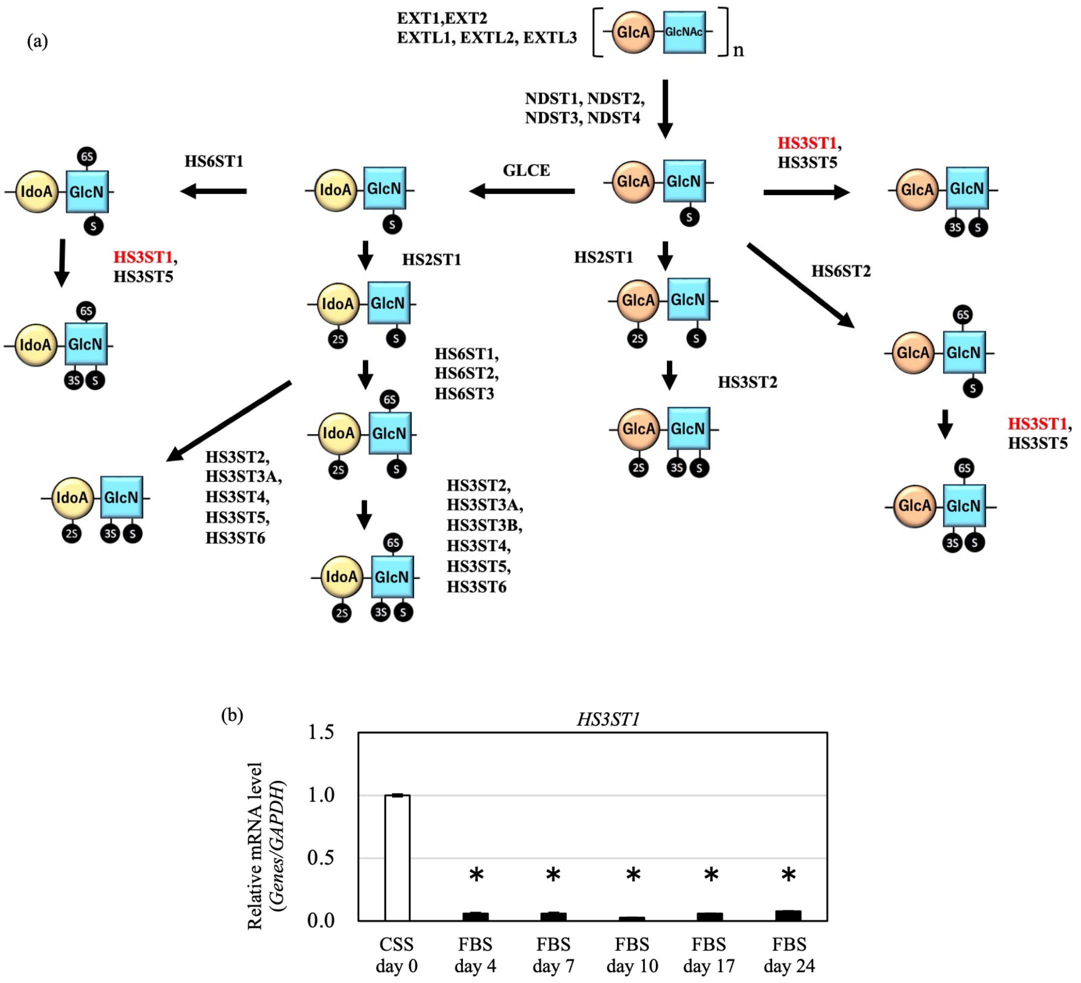

Supplementary Figure S2. *HS3ST1* is markedly decreased by switching to CSS-containing medium. (**a**) Synthetic pathway of heparan sulfate (HS). (**b**) mRNA levels of *HS3ST1* in C4-2 cells in cultured in medium containing FBS after pre-culture in CSS-containing medium for 24 days were analyzed by real-time PCR and normalized to *GAPDH* mRNA in the same sample. Expression levels are shown relative to gene expression at day 0. Ratios are given as mean ± S.E. of three independent experiments. Statistical significance assessed by Dunnett's test is indicated with *(p<0.01).

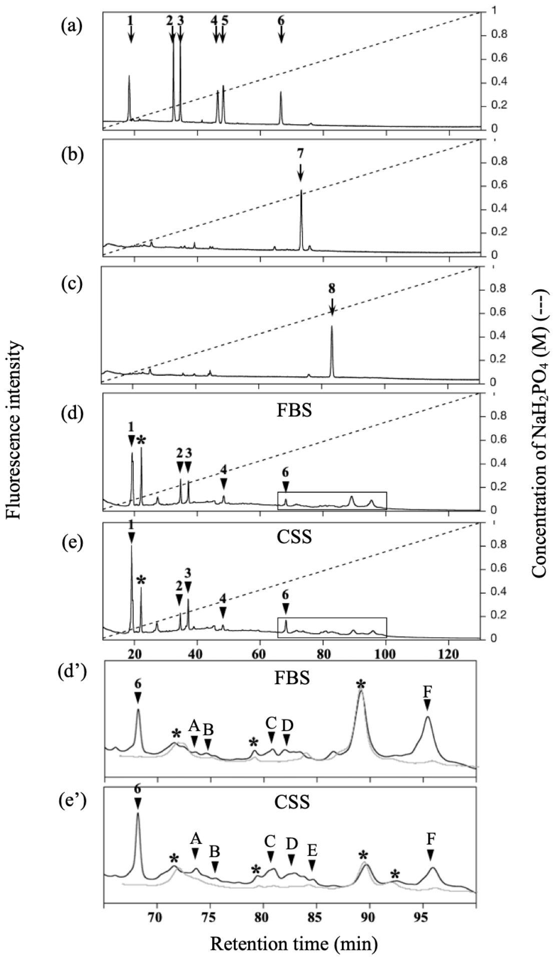

(f)

Heparinase-resistant oligosaccharides	C4-2 cells cultured in FBS containing medium	C4-2 cells cultured in CSS containing medium
	pmol disaccharide/mg total protein	
Peak A	0.3	2.8
Peak B	0.5	0.5
Peak C	2.0	3.2
Peak D	1.0	2.0
Peak E	ND	0.9
Peak F	26.7	20.4
Total	30.5	29.7

Supplementary Figure S3. HPLC profiles of the heparinase digest of GAG-peptides derived from C4-2 cells. (**a**) 2AB-labeled HS disaccharide standards. (**b**, **c**) 2AB-labeled structure-defined 3-*O*-sulfate-containing tetrasaccharides: ΔHexA-GlcNAc(6S)-GlcA-GlcN(NS,3S) (**b**) and ΔHexA-GlcNAc(6S)-GlcA-GlcN(NS,3S,6S) (**c**). (**d**, **e**) 2AB-labeled derivatives of di- and oligo-saccharides of GAG-peptides derived from C4-2 cells cultured in medium containing FBS (**d**) or CSS (**e**) after digestion with a mixture of

heparinase-I, heparinase-II, and heparinase-III. Derivatives were analyzed by anion-exchange HPLC on an amine-bound silica PA-G column using a linear gradient of NaH_2PO_4. (**d'**, **e'**) Enlargement (65–100 min) of **d** and **e**, respectively. The gray chromatograms in **d'** and **e'** are derived from substrate controls. The elution positions of authentic 2-AB-labeled HS standards are indicated by numbered arrows as follows: 1, ΔHexA-GlcNAc; 2, ΔHexA-GlcNAc(6S); 3, ΔHexA-GlcN(NS); 4, ΔHexA-GlcN(NS,6S); 5, ΔHexA(2S)-GlcN(NS); 6, ΔHexA(2S)-GlcN(NS,6S). Arrowheads (A–E) indicate the deduced heparinase-resistant oligosaccharides from prostate cancerous cells, which presumably contain GlcN(3S). Asterisks indicate impurities. (**f**) The amount of each oligosaccharide was calculated from the peak area in the chromatogram. ND, not detected (<0.1 pmol/mg protein).

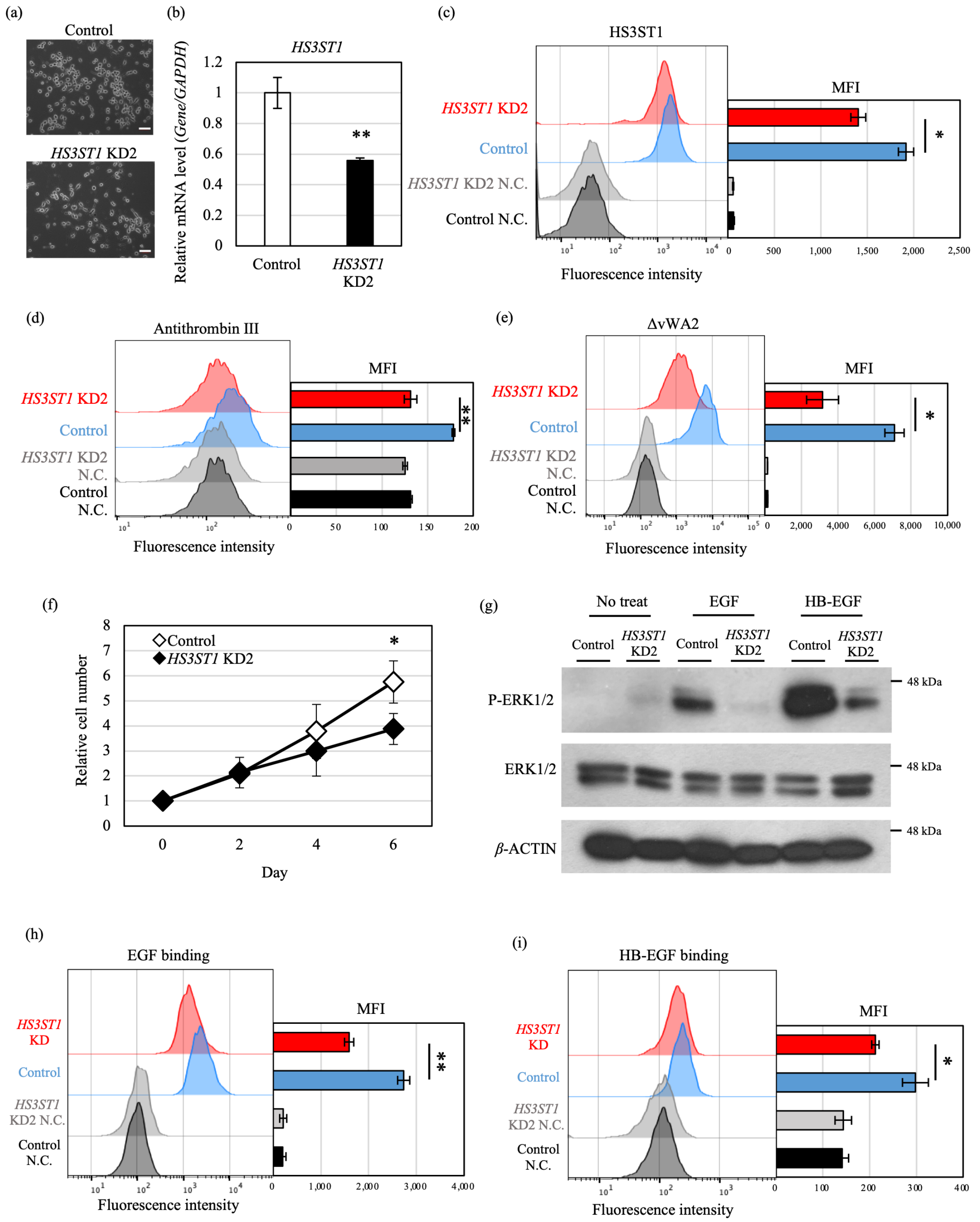

Supplementary Figure S4. Knockdown of *HS3ST1* using siRNA-2 also inhibits EGF and HB-EGF signaling and growth of C4-2 cells under hormone depletion. (**a**) Morphology of *HS3ST1* KD2 C4-2 cells cultured in medium containing CSS. C4-2 cells were transfected with *HS3ST1* siRNA, siRNA-2, and control siRNA three times every 3 days and cultured for 15 days in total. Scale bars, 100 μm. (**b**) Knockdown efficiency of

siRNA-2 was analyzed by real-time PCR. mRNA expression levels were normalized to *GAPDH* mRNA and are shown relative to the control. (**c**) Flow-cytometry analysis of HS3ST1 protein expression in *HS3ST1* KD C4-2 cells cultured in the medium containing CSS. Intracellular staining was performed with antibody against HS3ST1. Control and *HS3ST1* KD2 cells are indicated as blue and red, respectively. Negative controls reacted with only secondary antibody are indicated as black and grey. Right panel shows quantification of MFI. (**d**, **e**) Flow-cytometry analysis using antithrombin III and mutated cochlin ΔvWA2 in *HS3ST1* KD2 C4-2 cells in medium containing CSS. Control and *HS3ST1* KD2 cells are indicated as blue and red, respectively. Negative controls were reacted with only secondary antibody and are indicated as black and grey. (**f**) Cell proliferation assay of C4-2 cells transfected with control siRNA (◇) and *HS3ST1* siRNA-2 (◆) in medium containing CSS. Assay was started on 15th day after siRNA-2 transfection. (**g**) Western blot analysis of ERK1/2 phosphorylation after stimulation with 1 ng/ml of growth factor (EGF or HB-EGF) for 7.5 min. Before ligand stimulation, *HS3ST1* KD C4-2 cells were pre-cultured in serum-free medium for 24 hours on 15th day after siRNA-2 transfection. Each blot has been cropped from different gels; uncropped gels/blots are presented in Supplementary Fig. S12. (**h**, **i**) Flow-cytometry analysis of EGF and HB-EGF binding to *HS3ST1* KD2 C4-2 cells cultured in medium containing CSS. Control and *HS3ST1* KD2 cells are indicated as blue and red, respectively. Negative controls with no treatment are indicated as black and grey. Representative histograms from three independent experiments are shown. Ratios are mean ± S.E. of three independent experiments. Statistical significance assessed by Student’s t-test is indicated with *($p<0.05$) and **($p<0.001$).

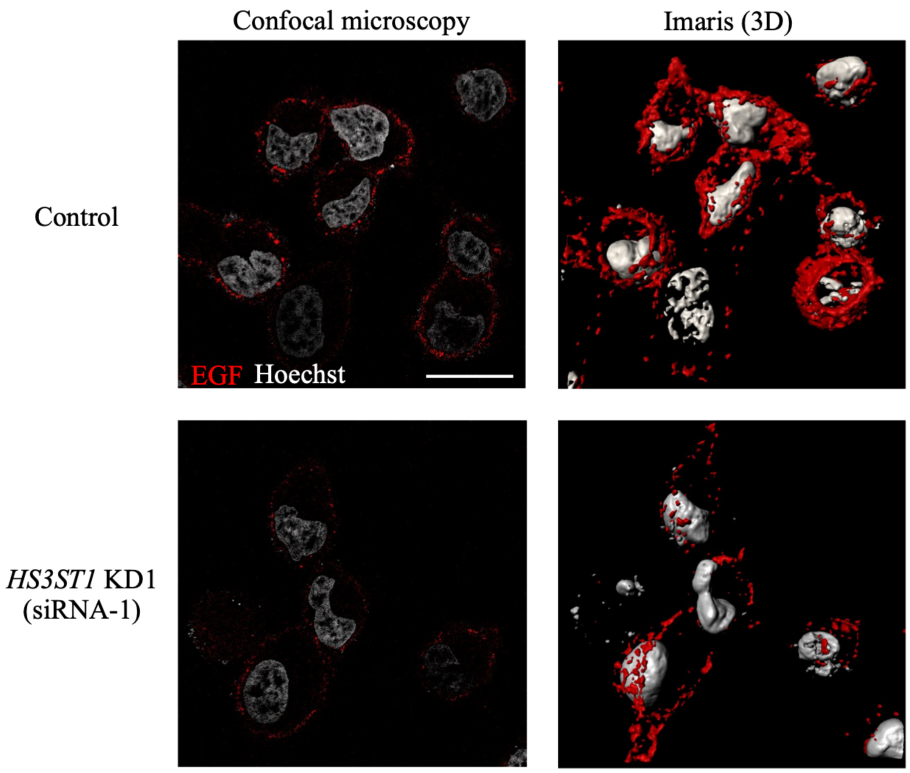

Supplementary Figure S5. EGF binding to the cell surface is decreased in *HS3ST1* KD cells. C4-2 cells were transfected with *HS3ST1* siRNA-1 and control siRNA. Cells were observed by confocal microscopy after stimulation with Alexa Fluor 647 conjugated EGF for 7.5 min. 3D images were created by Imaris. Scale bars, 20 μm.

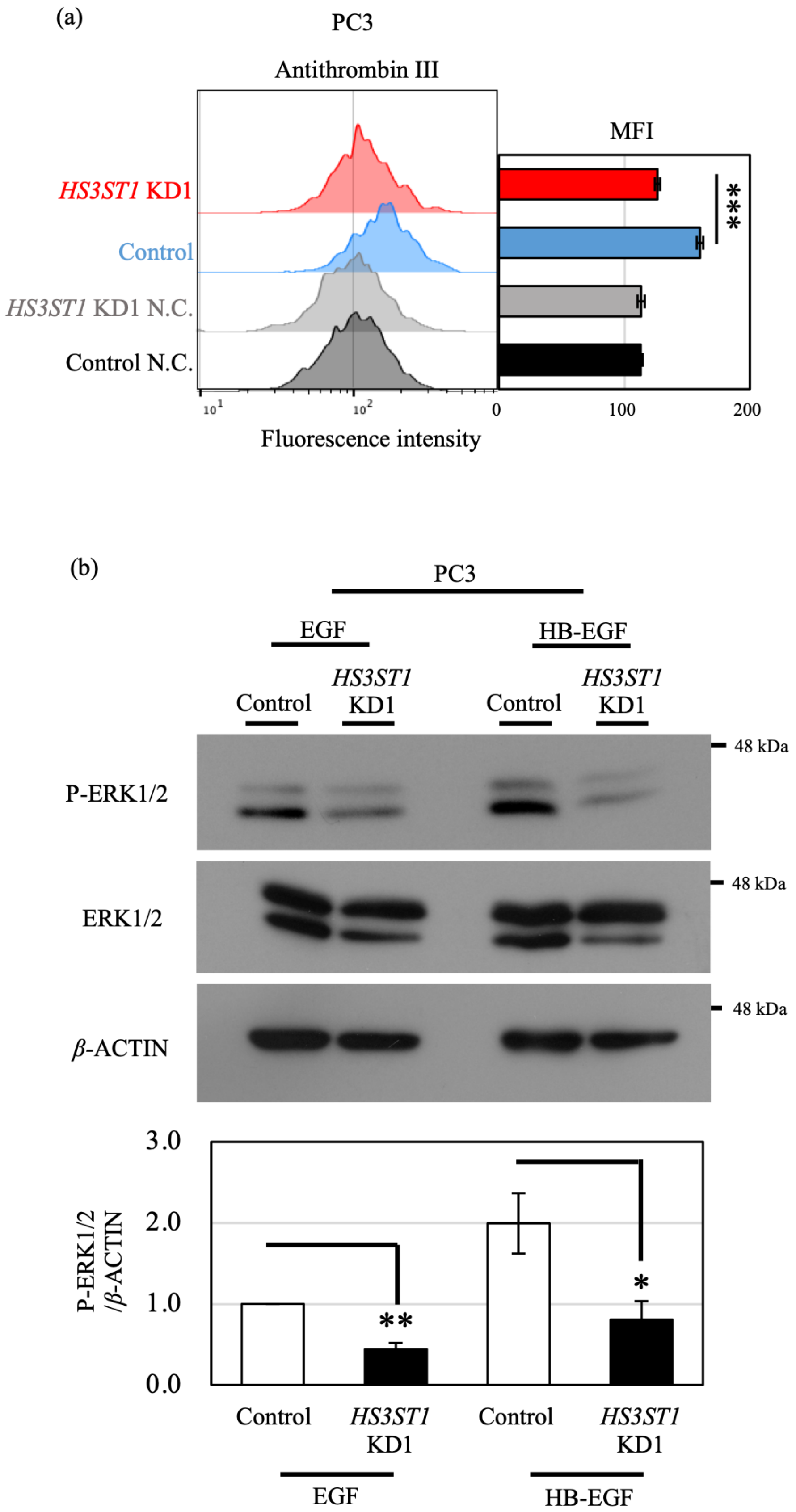
(a)
PC3
Antithrombin III
HS3ST1 KD1
Control
HS3ST1 KD1 N.C.
Control N.C.
MFI
***
0
100
200
Fluorescence intensity
(b)
PC3
EGF
HB-EGF
Control
HS3ST1 KD1
Control
HS3ST1 KD1
P-ERK1/2
ERK1/2
β-ACTIN
48 kDa
48 kDa
48 kDa
3.0
2.0
1.0
0.0
P-ERK1/2 /β-ACTIN
**
*
Control
HS3ST1 KD1
Control
HS3ST1 KD1
EGF
HB-EGF

Supplementary Figure S6. Knockdown of *HS3ST1* inhibits EGF and HB-EGF signaling in PC3 cells cultured in CSS-containing medium. (**a**) Flow-cytometry analysis using antithrombin III in *HS3ST1* KD PC3 cells. PC3 cells were cultured and transfected with siRNA in CSS-containing medium. Control and *HS3ST1* KD1 cells are indicated in blue and red, respectively. Negative controls, reacted with only secondary antibody, are indicated in black and grey. Right panel shows quantification of MFI. (**b**) Western blot analysis of ERK1/2 phosphorylation after stimulation with 1 ng/ml of growth factor for 7.5 min. Before ligand stimulation, cells were pre-cultured in serum-free medium for 24 hours on the 3rd day after siRNA-1 transfection. Bottom panel shows quantification of ERK1/2 phosphorylation normalized to β-ACTIN. Expression levels are shown relative to the control. Each blot has been cropped from different gels; uncropped gels/blots are presented in Supplementary Fig. S13. Ratios are given as mean ± S.E. of three independent experiments. Statistical significance assessed by Student's t-test is indicated with *($p<0.05$), **($p<0.01$) and ***($p<0.001$).

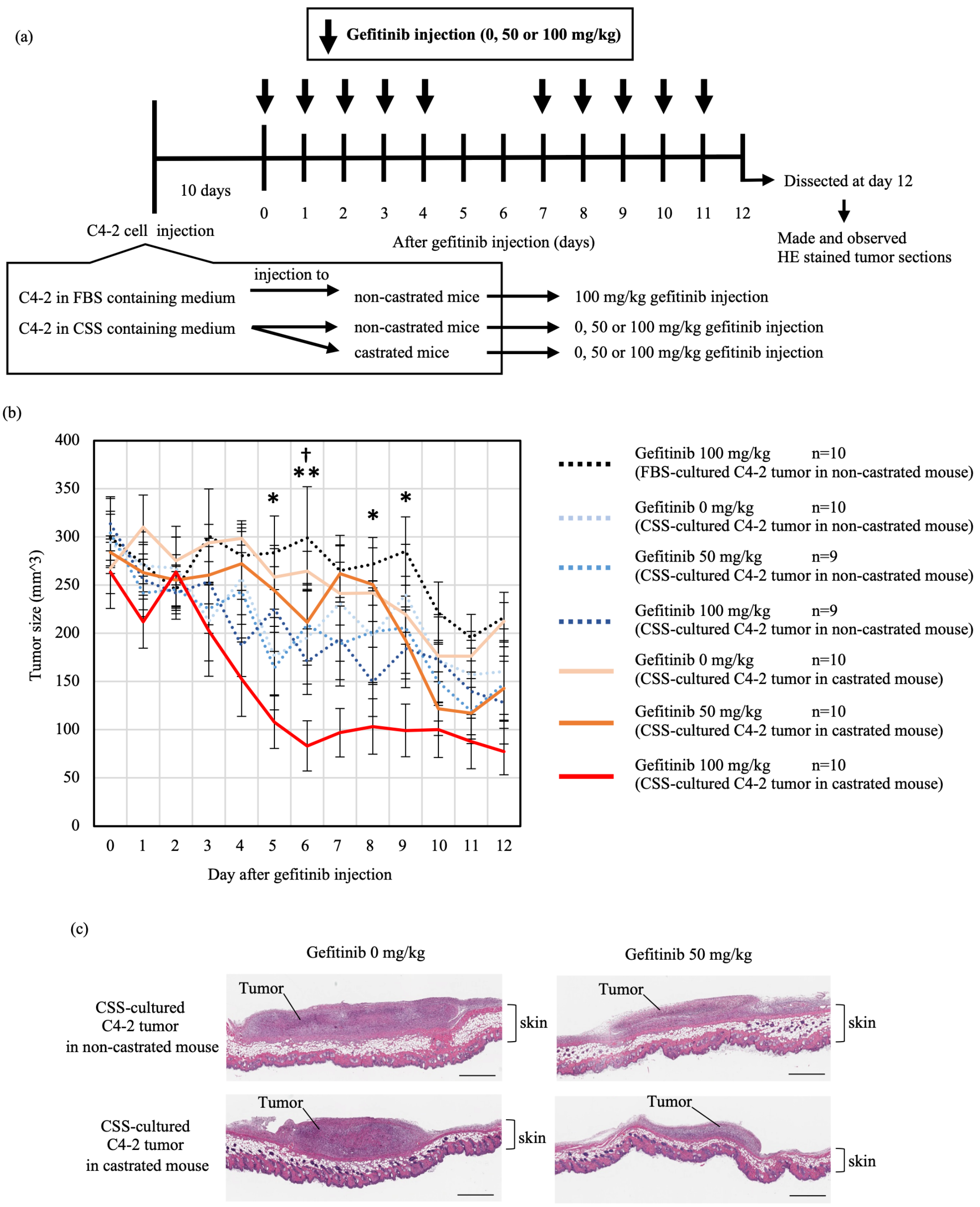

Supplementary Figure S7. Effectiveness of gefitinib on xenografted tumor in castrated mouse. (**a**) Scheme of gefitinib administration in mice with xenografted tumors. (**b**) Effects of gefitinib on growth of xenografted tumors in nude mice. Ratios are given as mean ± S.E. of 9 or 10 independent experiments. Statistical significance assessed by Tukey-Kramer test between 100 mg/kg gefitinib treatment for C4-2 tumors with pre-culture in FBS medium in non-castrated mice (black dotted line) and 100 mg/kg gefitinib treatment for C4-2 tumors with pre-culture in CSS medium in castrated mouse (red solid

line) is indicated as *($p<0.05$) at days 5, 8, and 9, and **($p<0.01$) at day 6. Statistical significance between 0 mg/kg gefitinib treatment for C4-2 tumors with pre-culture in CSS medium in castrated mouse (pale orange solid line) and 100 mg/kg gefitinib treatment for C4-2 tumors with pre-culture in CSS medium in castrated mouse (red solid line) is indicated as †($p<0.05$) at day 6. (**c**) Representative image of HE staining in xenografted tumor section. Scale bars, 800 μm.

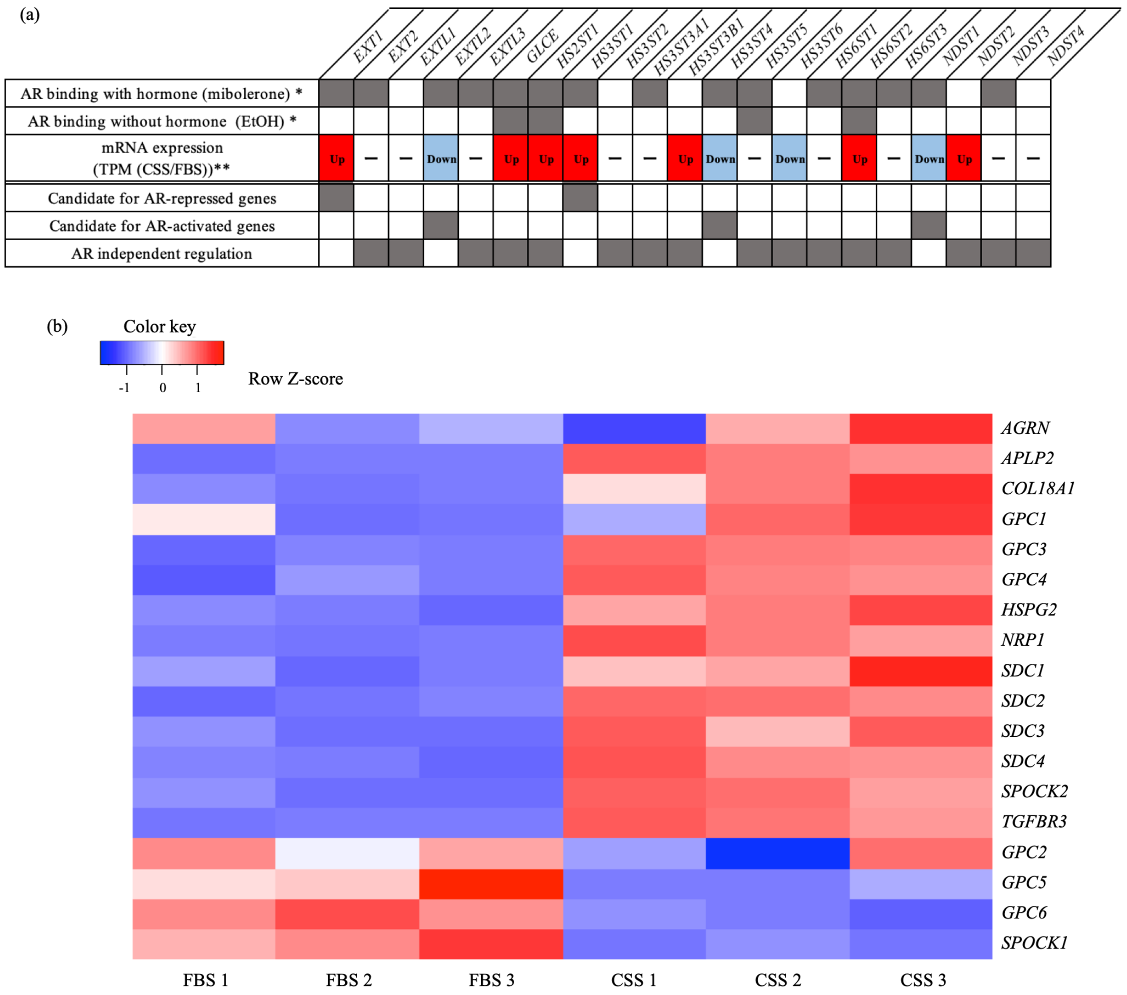

Supplementary Figure S8. Candidates for AR-repressed and AR-activated genes related to HS synthesis, and core proteins of 3-OS HS. (**a**) ChiP-Atlas analysis was used to determine whether AR binds to the genome of HS-related genes. *, Genes that AR bound in the presence of hormone (mibolerone) or the hormone-depleted (EtOH) condition are shown as grey columns (Ref. 46, 47). **, Fold change in mRNA expression in C4-2 cells cultured in CSS is also shown (see Fig. 2). Significantly upregulated genes in CSS culture versus FBS culture are indicated as red columns, and significantly downregulated genes as blue columns. In the lower lines, candidates for AR-repressed genes, candidates for AR-activated genes, and genes whose transcription activity seems to be regulated independently of AR are shown as grey columns. (**b**) HS proteoglycan expression profiles were extracted from RNA-seq data in C4-2 cells cultured in FBS- and CSS-containing medium.

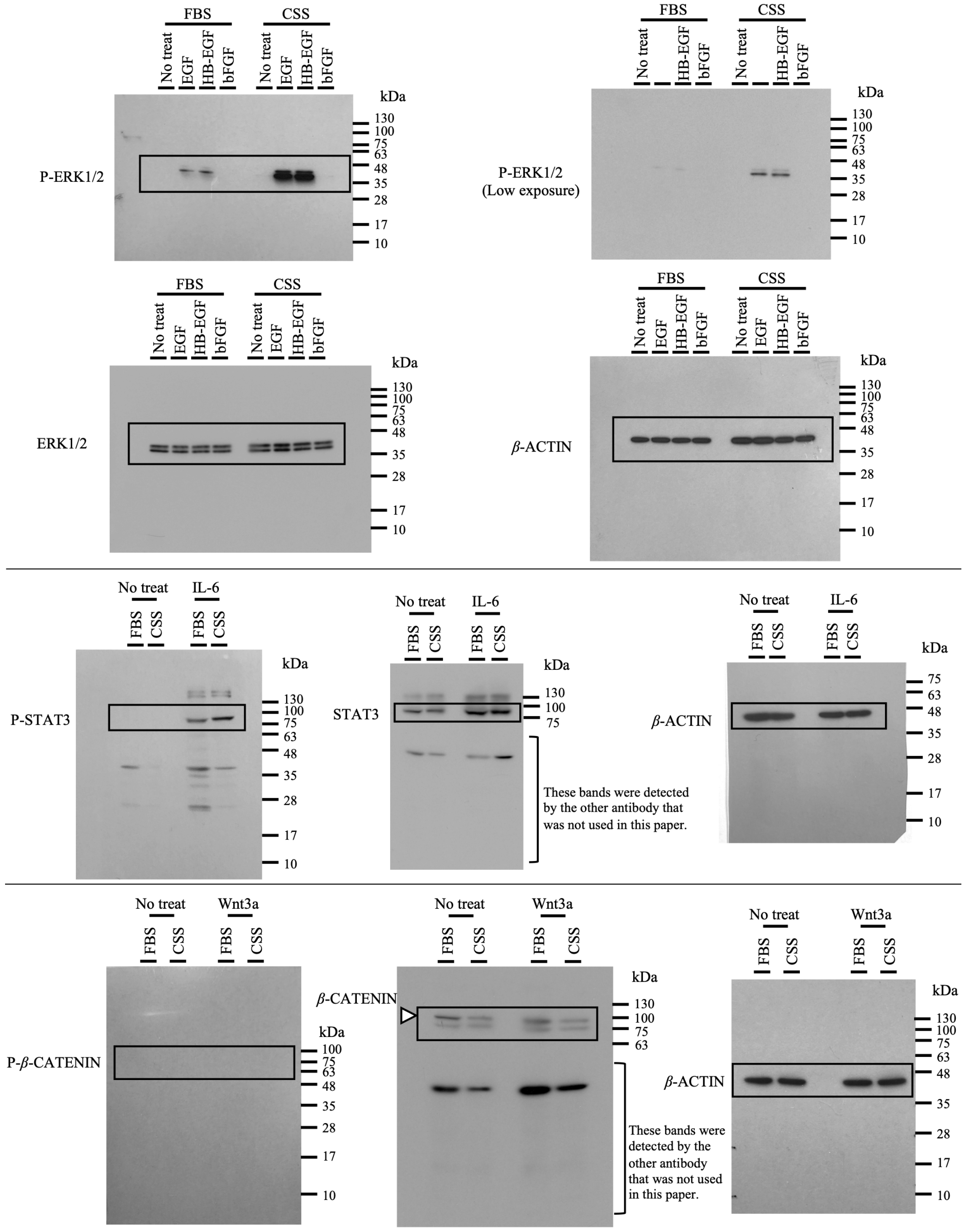

Supplementary Figure S9. Uncropped gels/blots related to Fig. 1.

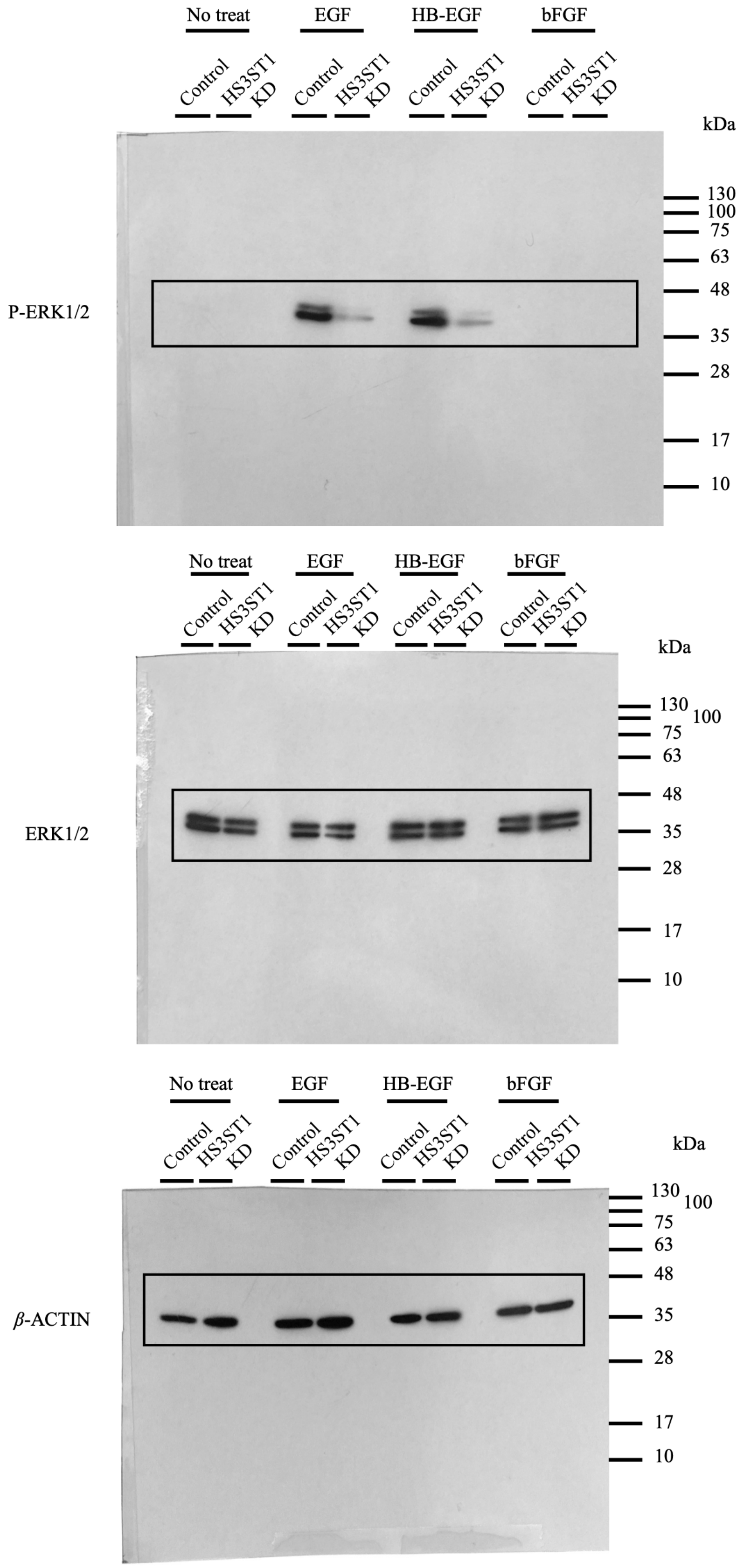

Supplementary Figure S10. Uncropped gels/blots related to Fig. 4.

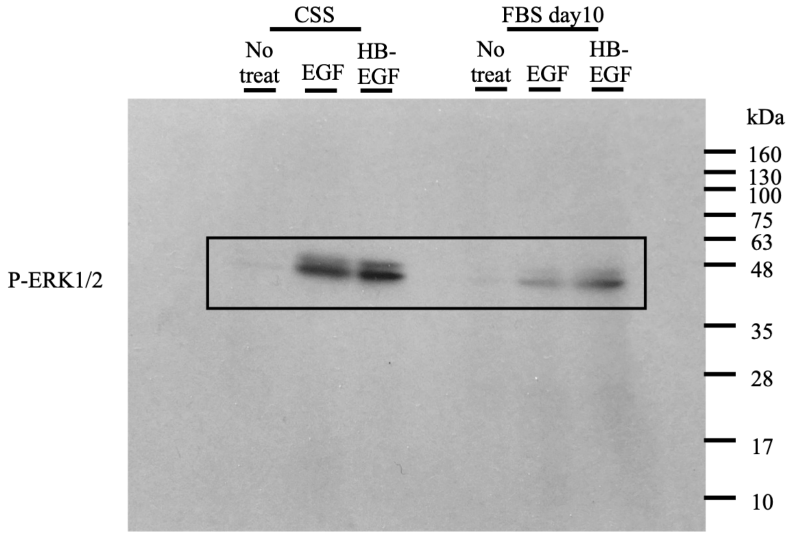

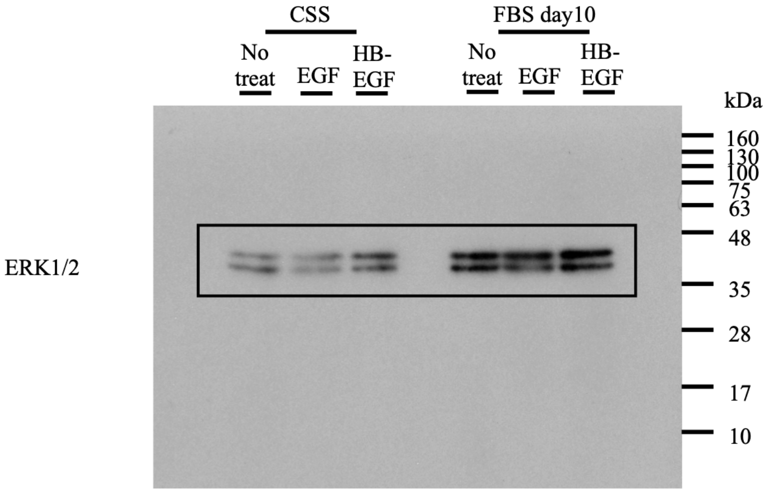

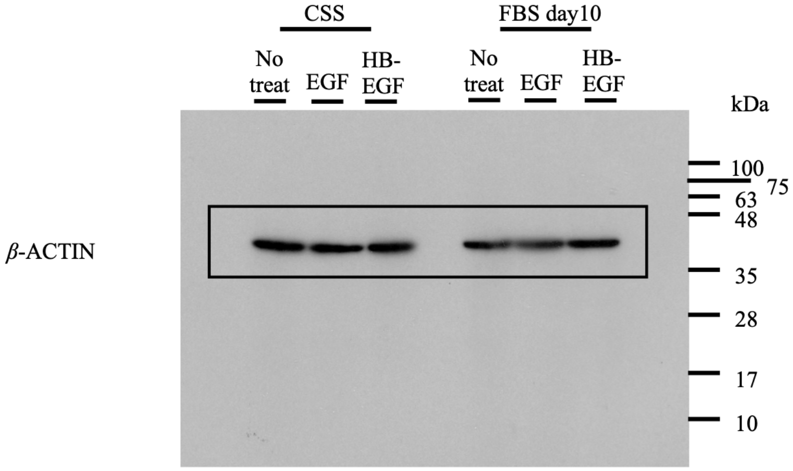

Supplementary Figure S11. Uncropped gels/blots related to Supplementary Fig. S1.

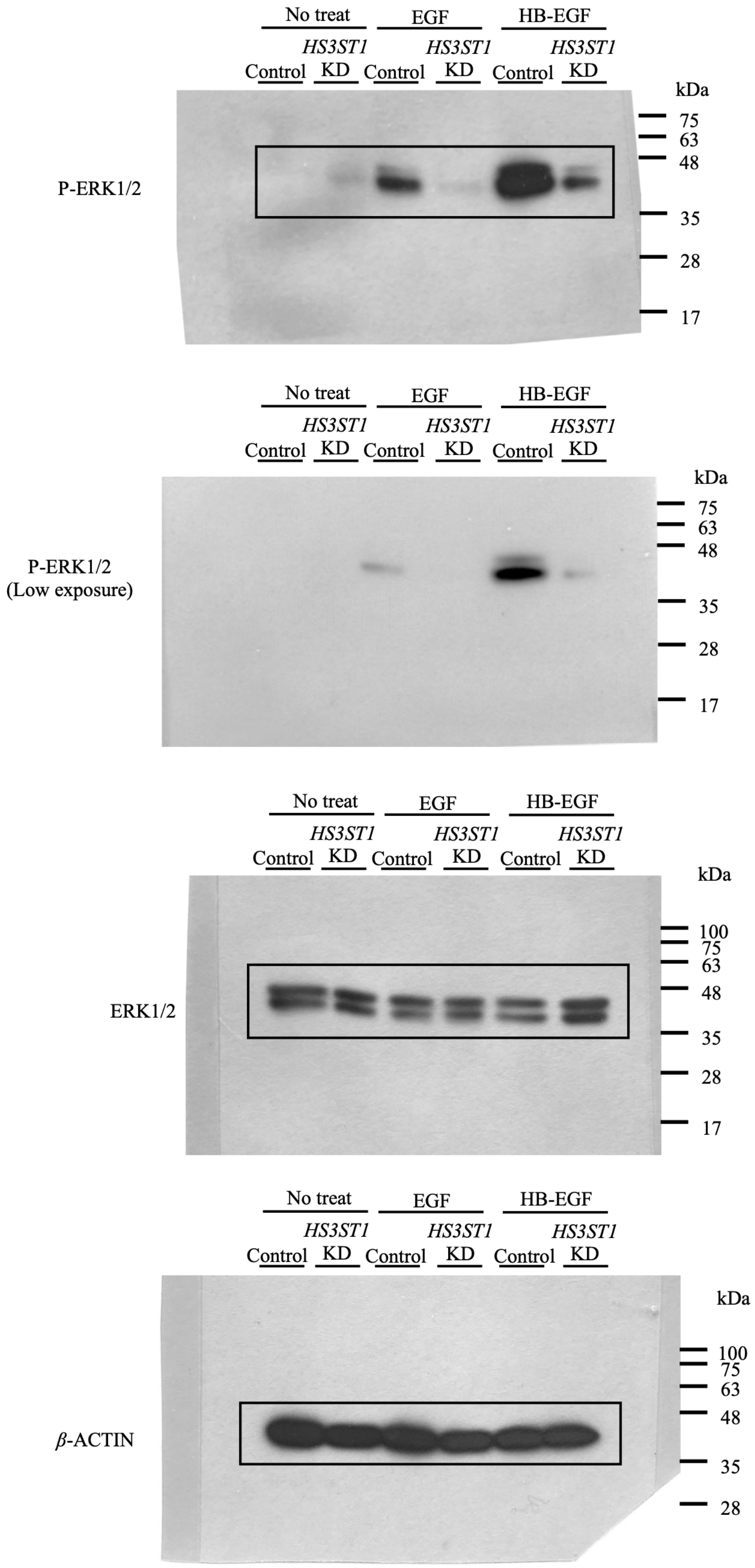

Supplementary Figure S12. Uncropped gels/blots related to Supplementary Fig. S4.

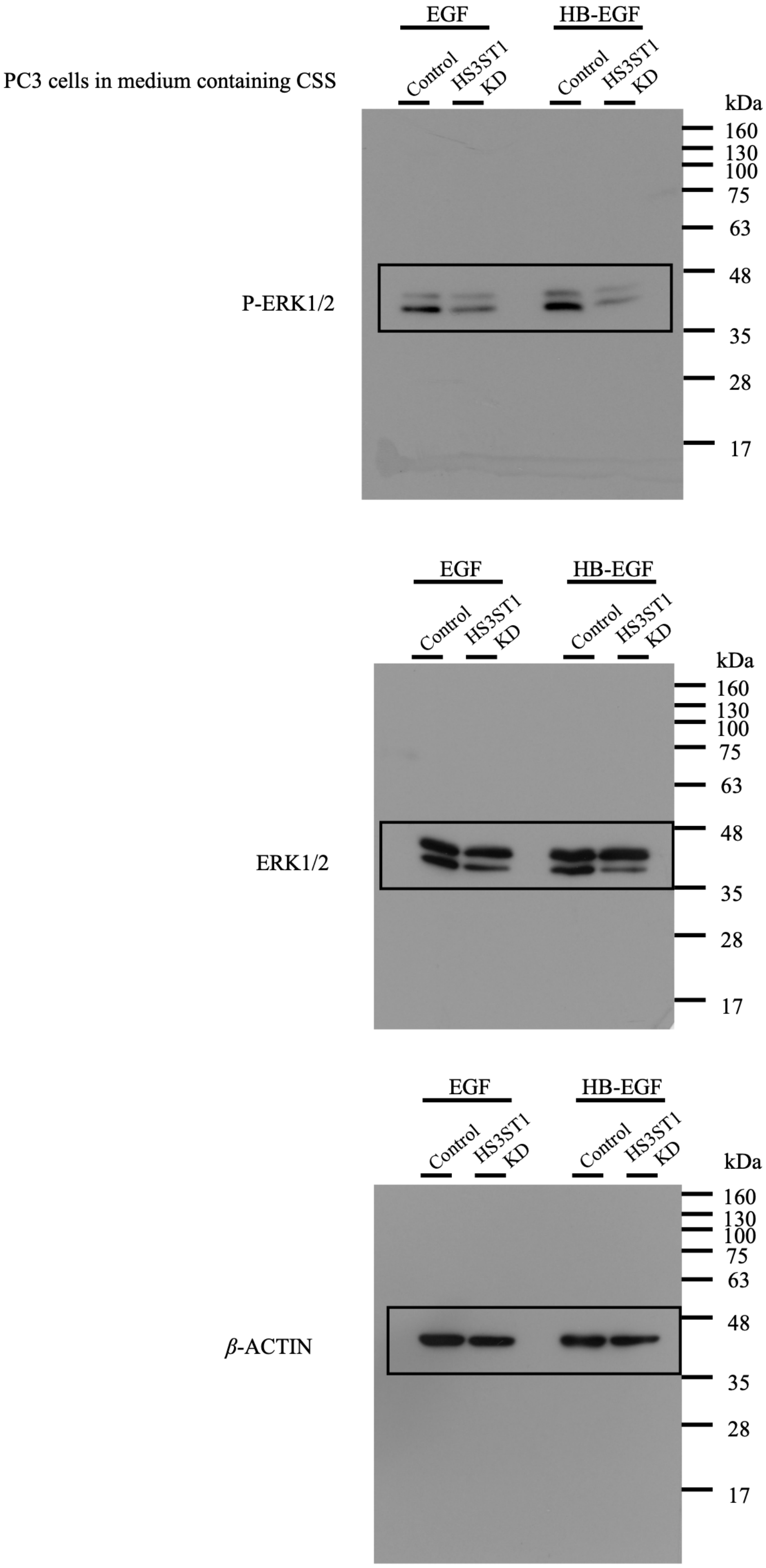

Supplementary Figure S13. Uncropped gels/blots related to Supplementary Fig. S6.

Supplementary Table S1. Measurement of tumor weight.

Supplementary Table S2. The primer sets used for real-time PCR.

Supplementary Table S3. The antibodies and molecules used for ligand stimulation (**a**), western blotting (**b**) and flow-cytometry (**c**).
